# Micropollutant concentration fluctuations in combined sewer overflows require short sampling intervals

**DOI:** 10.1016/j.wroa.2023.100202

**Published:** 2023-09-12

**Authors:** Viviane Furrer, Lena Mutzner, Christoph Ort, Heinz Singer

**Affiliations:** a Eawag, Swiss Federal Institute of Aquatic Science and Technology, 8600, Dübendorf, Switzerland; b Institute of Civil, Environmental and Geomatic Engineering, ETH Zürich, 8093, Zurich, Switzerland

**Keywords:** Trace contaminants, Combined sewer overflow, Sampling, Dynamics, Polar organic pollutants, High temporal resolution measurements

## Abstract

Combined sewer overflows (CSOs) are an important pathway of organic micropollutants from urban areas to open water bodies. Understanding the temporal dynamics of these micropollutants during overflow events is crucial for applying appropriate sampling methods and implementing effective management strategies. Yet, little is known about the dynamics of micropollutants in CSOs, because most studies report concentrations from single grab samples or event mean concentrations (EMCs). With unique high temporal resolution measurements (3 min), we show the real dynamics of polar organic micropollutants in CSOs of one small (2,700 people: P) and one large (159,000 P) urban catchment, for two micropollutant categories: (i) 33 micropollutants in municipal wastewater and (ii) 13 micropollutants from urban surface runoff. The concentration dynamics depend on the substance source and the catchment size. Indoor substances such as pharmaceuticals show high temporal dynamics with changes of 1 to 2 orders of magnitude within 9 min in the CSO of the small catchment. In contrast, outdoor substances at the small catchment and all substances at the large catchment display considerably lower variation. We tested various time-proportional sampling strategies to assess the range of error when estimating EMCs. We recommend an interval of 3 min to capture the dynamics of indoor substances in CSOs from small catchments. The results highlight that both future monitoring campaigns and the planning and management of urban wet-weather treatment systems will benefit from high temporal sampling resolutions, not only to understand dynamics but also to minimize errors of estimated EMCs.

## Highlights

• 3 min measurements reveal short-term dynamics of micropollutants in combined sewer overflow.
• Dynamics depend on the pollutant source and catchment size.
• Indoor substances in a small catchment show very high fluctuations.
• Outdoor substances fluctuate less than indoor substances.
• Short sampling intervals are required to capture these dynamics.

## Graphical abstract

**Figure:**
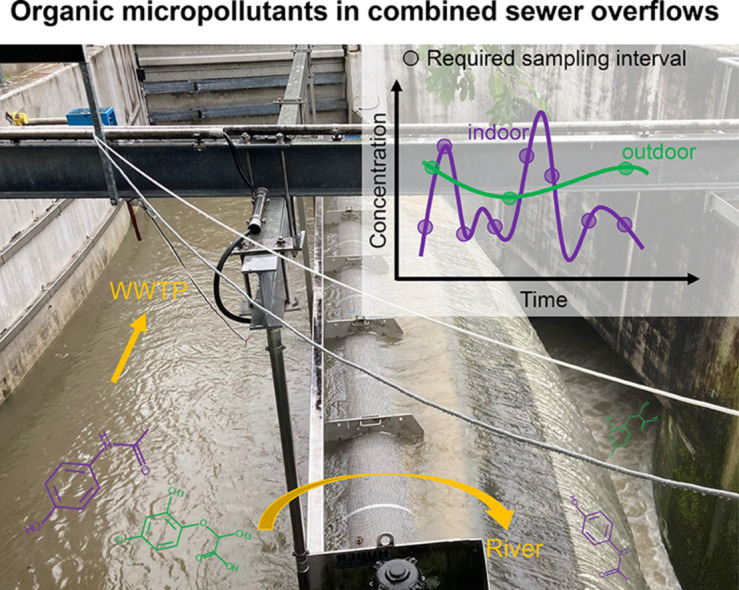

## 1 Introduction

Organic micropollutants emitted by urban drainage can harm the ecosystem and pose a risk to water resources (Gasperi et al., 2012; Launay et al., 2016; Nickel et al., 2021; Petrie, 2021). Combined sewer overflows (CSOs) are an important pathway for organic micropollutants to receiving water bodies (Musolff et al., 2010; Phillips et al., 2012). Over the last decade, modelling approaches (Mutzner et al., 2016) and monitoring data (Mutzner et al., 2022) have both shown that CSOs can exceed environmental quality standards for organic micropollutants.

Reliable monitoring data of organic micropollutants at CSOs is crucial for risk assessments and mitigation planning. The sampling strategy has to suit the concentration fluctuations of the parameters of interest (McCarthy et al., 2018; Ort et al., 2010b). To determine an appropriate sampling strategy, data must be monitored at short sampling intervals that reveal short-term variations. We assume that certain substances exhibit high dynamics in CSOs due to various former findings, as subsequently demonstrated. For example, high temporal resolution measurements of anthropogenic gadolinium, a contrast agent for magnetic resonance imaging, show a concentration increase of 40 times within 4 min in the inflow of a WWTP during dry conditions (Ort et al., 2010a). Also measured and modelled hourly concentration patterns of pharmaceuticals in sewers under dry conditions display strong concentration fluctuations (up to 10 × increase in 1 h) (Pouzol et al., 2020). It can be assumed that pharmaceuticals and personal care products (PPCPs) show similar if not even more pronounced concentration dynamics during rain events due to additional fluctuation from varying dilution. This is supported by a study by Madoux-Humery et al. (2013), who measured four PPCPs – caffeine, carbamazepine, theophylline and acetaminophen – at high temporal resolution (5 to 30 min) and found concentration changes ranging from one to two orders of magnitude during a rain event (up to 10 × decrease in 10 min). Additionally, pesticides in wet-weather discharges of urban catchments can also display substantial concentration fluctuations, although with less rapid changes (up to 10 × increase in 2 h), as demonstrated for mecoprop and atrazine by Wittmer et al. (2010).

Overall, data is lacking on the short-term dynamics of organic micropollutants in CSOs, as monitoring campaigns report mainly event mean concentrations (EMCs). Of 29 past studies on polar organic micropollutants in wet-weather discharges, 30 % collected as little as a single grab sample per event (Spahr et al., 2020). Moreover, no study has yet been conducted that demonstrates concentration fluctuations at high temporal resolution for a large suite of compounds with different use patterns. However, high-resolution measurement data capturing the dynamics of organic micropollutants in wet-weather discharges are needed to design a suitable sampling strategy, which is crucial for reliable risk assessment and urban pollution management.

We aim to address this gap by providing high temporal resolution measurements for a broad range of organic micropollutants (33 indoor and 13 outdoor substances) from two CSO sites with very different population and catchment sizes (A: 2,700 people: P, 17 ha_red_; B: 159,000 P, 368 ha_red_). We took 3 min grab samples for a period of one hour and complemented them with continuously pumped 3 min composite samples to identify instances where fluctuations might be even higher than 3 min. With this approach, we sampled three events at the CSO in catchment A and one event in catchment B. The samples were analysed with liquid chromatography high-resolution mass spectrometry (LC-HRMS). We used this dataset to test various time-proportional sampling strategies. The goals of this study are to (1) investigate the short-term concentration fluctuations of substances from different sources, (2) examine the influence of the catchment size on pollutant dynamics, and (3) help to choose an optimal sampling strategy for future monitoring campaigns.

## 2 Results and discussion

### 2.1 High temporal resolution measurements

#### 2.1.1 High-resolution time series

**Fig. 1:**
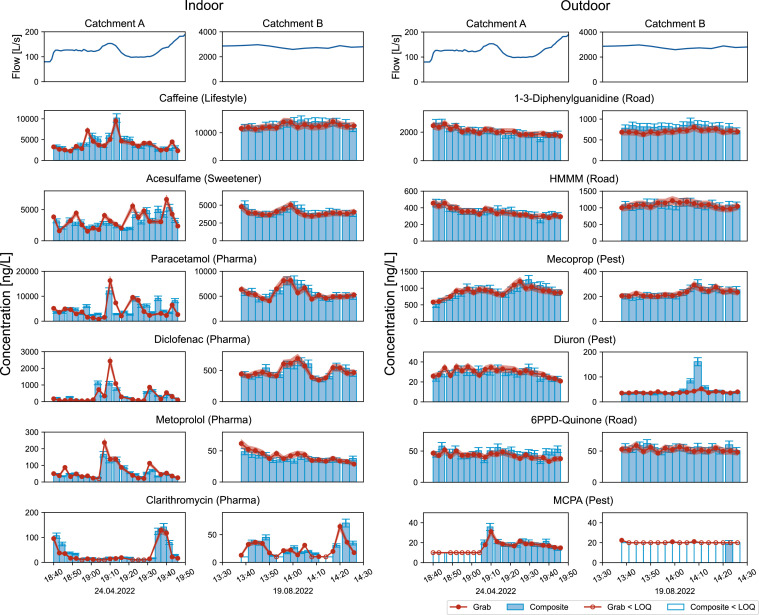
Flow (inflow to CSO) and concentration (with 10 % error band) of indoor (left) and outdoor (right) substances from 3 min grab (red dots) and 3 min composite samples (blue bars) of overflow event from 24.4.2022 in catchment A (2,700 P) and event from 19.8.2022 in catchment B (159,000 P). Empty bars and points represent concentrations lower than level of quantification (LOQ). Pharma = Pharmaceuticals, Pest = Pesticides.

The short sampling intervals reveal pronounced variability in concentrations of organic micropollutants during CSO events. Fig. 1 shows an example set of substances from indoor and outdoor applications for one event in each catchment (for all substances and events, see SI Section 2). The concentrations from both the 3 min grab and continuously pumped 3 min composite samples are depicted to demonstrate the results from two independent sampling methods and to indicate the ability to capture the true dynamics with a sampling resolution of 3 min.

The highest fluctuations can be observed for indoor substances at the small catchment A. The indoor substances at the large catchment B, and the outdoor substances in both catchments show much less variation.

At the CSO in catchment A, indoor substances exhibit short peaks lasting only 3 to 6 min. In contrast, the indoor substances in catchment B show much less variation.

Outdoor substances at both CSOs show constant or slightly decreasing concentrations with the exception of the pesticide diuron, which exhibits a 6 min peak in the composite samples at catchment B.

#### 2.1.2 Quantitative description of fluctuations with flashiness index

**Fig. 2:**
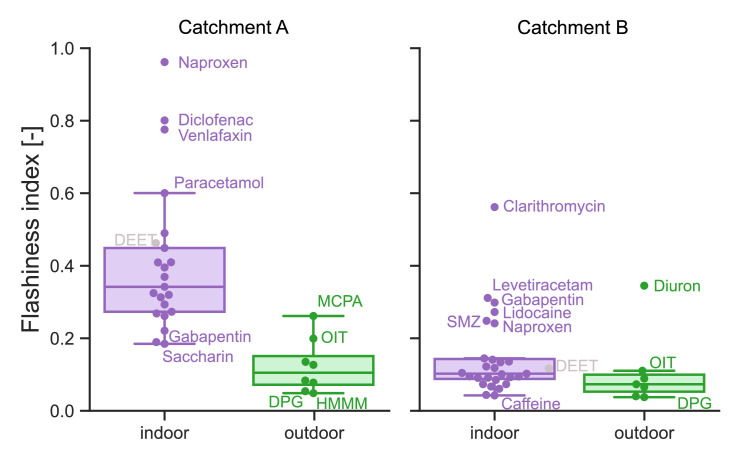
Boxplot of flashiness index of 3 min composite samples for all substances, distinguished between indoor and outdoor application (DEET (grey) has indoor and outdoor applications). Left: catchment A (2,700 P). Right: catchment B (159,000 P). DPG = 1-3-Diphenylguanidine, SMZ = Sulfamethoxazole.

To describe and compare the fluctuations of the concentration time series (Fig. 1) quantitatively, we calculated the flashiness index (see Section 4.6). Higher concentration fluctuations result in higher flashiness indices. As can be seen in Fig. 2 for catchment A, indoor substances exhibit higher flashiness indices (median_A_indoor_: 0.37) than outdoor substances (median_A_outdoor_: 0.11). However, the difference in flashiness indices in catchment B (median_B_indoor_: 0.10, median_B_outdoor_: 0.08) is not as apparent.

To compare the variation in observed fluctuations across different events, we monitored three events at catchment A because we expected higher inter-event differences in the smaller catchment due to higher dynamics. The corresponding timeseries and flashiness indices are shown in SI Section 2.2 and 3.2. Interestingly, the flashiness indices exhibit very similar distributions in each substance group for all three events in catchment A, indicating that sampling one event per site is probably sufficient to capture the ranges of dynamics of all substances combined. However, when observing single substances, interevent differences might be larger because temporal factors such as seasonal application and daytime might influence the concentration levels and dynamics substantially.

#### 2.1.3 Explanation for observed dynamics

**Indoor substances.** The higher fluctuations of indoor substances than outdoor substances can be explained by characteristics of their sources. Indoor substances enter the combined sewer system mainly through pulsed flushes from toilets, dishwashers, and water taps, which create short peaks (10 – 30 sec) on entry. Each concentration peak widens along the flow path due to dispersion effects, which can be substantial in large catchments (Rieckermann, 2005). The concentration dynamics of indoor substances also depend on the number of people excreting them, as a larger number of point sources results in overlapping effects that attenuate concentration changes (Pouzol et al., 2020). This can be observed in Fig. 1: for example caffeine, which is consumed by many people, shows much lower fluctuations in both catchments than the antibiotic clarithromycin, which is likely taken by only very few individuals, particularly in catchment A. The effect can also be observed when comparing each substance across the catchments: less fluctuation is observed in catchment B, where more point sources overlap.

Tolouei et al. (2019) showed that even during wet-weather periods, daily patterns of indoor substances in raw wastewater are strongly driven by human activities. Furthermore, Madoux-Humery et al. (2013) measured the PPCPs caffeine, carbamazepine, and acetaminophen at a temporal resolution of 5 min for the first 15 min and subsequently 30 min at a CSO in a catchment with 20,000 People Equivalents (PE). The results reveal concentration changes ranging from one to two orders of magnitude during a rain event. For caffeine and acetaminophen, we observe similar changes to Madoux-Humery et al. (2013). During rain events, the dynamics of indoor substances are driven by concentration changes in the wastewater and are further influenced by the variable dilution factor due to varying stormwater flow.

**Outdoor substances.** Substances that are applied outdoors are washed off by rain from surfaces such as roads, flat roofs, facades, and green areas, where they accumulated during dry periods or are leached out from. Little is yet known about their dynamics in wet-weather discharges due to lack of high temporal resolution data. Peter et al. (2020) demonstrated that pollutographs of stormwater driven organic micropollutants in a small urban stream deviate from storm hydrographs. Instead, they exhibit a rapid concentration increase during the first phase, when the discharge in the stream is still low, followed by elevated concentrations throughout the rain event. This indicates transport-limited wash-off and leach-out processes caused by large reservoirs of contaminants leading to water contamination not only during the first flush but rather during the entire runoff event. This aligns with our measurements: outdoor substances show rather constant concentration over the 1h sampling period in both catchments. In line with our findings, studies conducted by Burkhardt et al. (2011) in a small Swiss catchment (site 1: 4 buildings, 0.5 ha; site 2: 11 ha) and Bollmann et al. (2014) in a Danish catchment (140 houses, 7.1 ha_red_) have shown that the leaching of biocides from facades does not exhibit a first flush during rain events but remains constant throughout a rain event. Wittmer et al. (2010) observed different concentration dynamics in emissions of pesticides from urban and agricultural areas depending on the compounds and sources. The substances applied outdoor showed rain-event-driven peaks that were either seasonal (agriculture & urban) or throughout the year (urban) depending on the application. Wittmer et al. (2010) observed a maximum concentration change of 10 × within 2 hours. We also see an increase of 8 × within 6 minutes for MCPA (Fig. 1). For other pesticides, we observe more constant concentrations over the 1-hour sampling period. Furthermore, pesticides can also be applied indoors or be disposed inappropriately through the sink, which can lead to higher dynamics than observed here.

The classification of indoor and outdoor is not always straightforward, for example with DEET (concentration in SI Section 2, flashiness index in Fig. 2). DEET exhibits a wide range of uses, including human and animal insect repellents, incorporation into textiles, and industrial applications. For such substances, the application with the highest dynamics has to be identified to determine the optimal sampling frequency. When monitoring several substances, it is important to choose the sampling interval for the substance with the highest variability.

Overall, we found that the strong fluctuations of indoor substances align quite well with previous studies and can be explained by the number of people consuming the substance and the catchment size. Most outdoor substances in our measurements exhibit less variable concentrations, which is in line with earlier research that suggests transport-limited wash-off processes as the main driver of the dynamics of outdoor substances. However, we also see some exceptions in which outdoor substances show stronger dynamics, such as MCPA and imidacloprid.

#### 2.1.4 Real concentration fluctuations

To estimate whether the dynamics of the substances investigated could even be higher than 3 min, we compared the 3 min grab and 3 min composite samples. The disparity between the two sampling methods provides a rough estimation of the real concentration fluctuations, assuming that the difference between the two sampling strategies should be smaller than the uncertainty due to chemical analysis. For substances with small differences, it is assumed that the real dynamics do not exceed 3 min and that the measured data reflects the concentration variations accurately. The calculated differences are highest for indoor substances in the small catchment (see SI Section 4). It is difficult to define a specific threshold above which fluctuations seem to exceed 3 min. However, visual assessment of the time series and comparison with corresponding differences reveal that substances with a difference exceeding 60 %, such as naproxen, tramadol, venlafaxin, ibuprofen, diclofenac, and paracetamol in catchment A, may exhibit fluctuations below 3 min. In general, such high dynamics are to be expected for substances with a pulsed entry into the sewers (e.g. toilet flush) in small catchments where the number of entries and mixing and dispersion effects are small. To uncover such fluctuations, even higher sampling frequencies would be needed, which exceed the sampling capacities currently available.

### 2.2 Testing various sampling strategies

To investigate the influence of longer sampling intervals on the correctness of the estimated EMC, we tested a range of time intervals (6, 9, 15, 30, 60 min) on the 3 min composite samples. For every substance, we calculated the maximum over- and underestimation of EMC for every time interval compared to the ‘true’ EMC, calculated as reference from the 3-minute data.

**Fig. 3:**
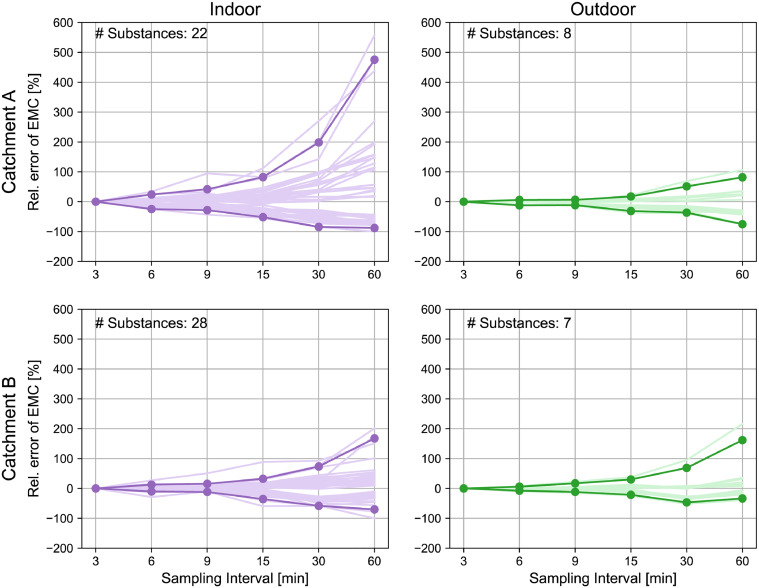
Maximum relative error of event mean concentration for each substance (light-coloured lines) and 95 % quantile across all substances (dark lines) for various sampling intervals for indoor (left) and outdoor (right) substances, for catchment A (top, 2,700 P, event from 24.04.2022) and catchment B (bottom, 159,000 P, event from 19.08.2022). Zoomed y-axis can be found in SI Section 5.

The results are presented in Fig. 3, which highlights the differences between the catchment sizes and substance groups. The indoor substances in catchment A show the highest relative error in the estimated EMC with a 95 % quantile of 580 % at a sampling interval of 60 min. The other three cases show similar errors of around 200 % at a sampling interval of 60 min. This can be attributed to the flashiness index (Fig. 2), which demonstrates that the higher the fluctuations, the greater the error in the estimated EMC. Here, we choose a 95 %-quantile band to demonstrate the worst-case errors. If one is only interested in single substances such as acesulfame that vary much less than average, the resulting error would be smaller.

With longer sampling intervals, the real EMC is overestimated stronger than underestimated (relative error_A_indoor_60min_: 580 % vs. –95 %). This can be explained by the fact that our data only comprises positive concentrations, limiting the maximum potential underestimation to –100 %. In contrast, overestimation is contingent on the variance between peak and mean concentrations, which can exceed 100 %. However, despite the higher magnitude of overestimation, underestimation occurs more frequently, as evident from the analysis of all possible outcomes for one substance (see Fig. 5). This can be explained by the fact, that the micropollutant time series show peaks rather than valleys. Consequently, the probability of missing the peak concentrations is higher than of sampling it. This underscores the importance of a shorter sampling interval to ensure a conservative risk assessment.

The increase in relative error of the EMC with longer sampling intervals follows an exponential trend. Hence, choosing an appropriate sampling interval is crucial because even a slight decrease in the sampling frequency can result in a significant increase in error. Moreover, even with short sampling intervals, the error can still be substantial. For instance, some indoor substances such as naproxen, tramadol, and venlafaxin in the catchment A display more than 70 % error with a 15 min sampling interval.

To minimize the sampling uncertainty, a continuously pumped sample would be ideal, but this increases the risk of clogging due to the constant pumping of combined sewage that contains solids. In practice, taking subsamples every 2 min (minimal pumping interval of our automated sampler) for composite samples has proven effective for unsupervised sampling over several hours without clogging as long as some basic clogging prevention measures are in place, such as a protective shield. Thus, when a palette of indoor and outdoor substances is measured, sampling intervals of a few minutes are recommended to reduce the error of EMCs to below 50 %.

Selecting a minimal sampling interval also accounts for other factors that could potentially result in even higher dynamics than our current data indicates: (1) pharmaceuticals that are consumed by a small portion of the population but exhibiting high excretion concentrations, such as x-ray contrast media; (2) unintended disposal as for example pharmaceuticals through toilets; or (3) strong first flush phenomenon, where outdoor substances exhibit higher concentrations at the start of a rain event due to a limited source (e.g. pesticides).

The concentration measured at the CSO may differ from the concentration at the source for certain substances that decay within the sewer system. However, the decay process should not affect the variability of the signal, so our findings are also applicable to unstable substances.

#### 2.2.1 Transferability to other sites

The catchment sizes investigated in this study highlight the difference between a smaller and larger catchment. However, there are catchments with even smaller areas and fewer connected people than catchment A. Extrapolating our findings to these smaller catchments is challenging. It can be assumed that indoor substances in smaller catchments exhibit higher dynamics than those found in our study due to the reduced number of point sources and smaller dispersion effects. However, formulating an assumption for outdoor substances is difficult, and more investigation is needed. Larger catchments are likely to demonstrate even less variation, thereby diminishing the risk of underestimation of the required sampling interval from our measurements.

The dynamics of outdoor substances we observed may potentially apply to stormwater overflows in separate sewer systems. However, certain differences could impede the transferability of our findings from combined sewers: (1) even small precipitation events typically lead to stormwater overflows, which may cause different intra-event dynamics, such as a more pronounced first flush, and (2) smaller catchments and thus shorter flow durations lead to less dispersion and fewer mixing phenomena. Hence, sampling campaigns specific to stormwater overflows would benefit from checking high-temporal wash-off dynamics.

## 3 Conclusion

The high temporal resolution measurements of organic micropollutants at CSOs in a small and a large catchment revealed the following:

• Organic micropollutants show different degrees of fluctuations in CSOs, ranging from substantial variation within a few minutes to more constant concentration profiles over more than 30 min. The data suggests two major factors that influence the dynamics: (1) the sources of micropollutants, where indoor substances tend to exhibit higher fluctuations than outdoor substances, and (2) the catchment size, where smaller catchments exhibit more pronounced dynamics due to greater variability in sources and smaller effects of dispersion and mixing.
• We observe high fluctuations of indoor substances at the small catchment, which in some cases even exceed the 3 min sampling interval. Therefore, we recommend a 2 min sampling interval if a diverse range of substances are included in the monitoring campaign to minimize uncertainty while remaining technically feasible. Uncertainties can also be minimized through continuously pumped composite samples, but this may lead to clogging problems when sampling combined sewage. If a 2 min interval cannot be achieved, the resulting error in EMC can be estimated as in Fig. 3. Representative samples are crucial for conducting thorough risk assessments and planning mitigation measures.
• The pronounced dynamics of organic micropollutants in CSOs shown in this paper emphasize the importance of not only sampling but also analysing at high frequency to gain deeper insights into concentration and load variations. By doing so, future research can address crucial questions concerning risk assessment and management of CSOs.

## 4 Materials and methods

### 4.1 Study sites

**Catchment A.** The first investigated CSO (CSO A) lies in a rural catchment 15 km east of Zürich in the village of Russikon, Switzerland (coordinates: 47°23′29.253″N 8°46′12.112″E). The catchment encompasses a typical Swiss rural village with households and green areas surrounded with agricultural fields. It has an effective hydraulic area of 17 ha_red_ and 2,700 P are connected to the combined sewer system. The catchment is defined as the entire connected upstream area, irrespective of CSOs located further up the network. The land use is predominantly households and roads, with agriculture on the outskirts of the urban area and little industry. The maximum hydraulic retention time to the sampling site is 15 min. CSO A has a storage capacity of 280 m^3^ (16 m^3^/ha_red_), which is arranged as a catch basin (230 m^3^) that stores the first volume of an overflow event and an additional 50 m^3^ that are retained in the channel. The outlet to the WWTP is limited to 80 L/s. The excess flow is discharged through a side weir into a nearby stream. Detailed schemes of both CSOs can be found in SI Section 6. Samples were collected at the inflow channel upstream of the side weir, 60 cm above the channel bottom.

**Catchment B.** The second sampling site lies in a much larger catchment with 368 ha_red_ and 159,000 P (184,000 PE) in the canton of Zug, Switzerland. This catchment consists primarily of households, roads, and agriculture, with a small quantity of industry. The CSO investigated (CSO B1) is located in the inflow channel to the WWTP Schönau (coordinates: 47°11′50.509″N 8°26′33.923″E). If the WWTP inflow exceeds 2,500 L/s (Q_dry_weather_ = 300 – 800 L/s), the combined sewer is discharged through a side weir into a middle-sized river (Q_dry_weather_ = 50 m^3^/s). Sampling was conducted at the upper end of the side weir, in the middle of the channel and 0.8 m above the channel bottom, enabling the collection of samples at flow rates above 1,500 L/s. The maximum hydraulic retention time to the sampling site is 5 h. A second CSO (CSO B2) is located after the grid chamber at the WWTP with a storage basin of 3,000 m^3^ (8 m^3^/ha_red_), which is filled when the WWTP inflow discharge exceeds 1,600 L/s. CSO B1 was selected for sampling to be able to capture the high dynamics of micropollutant concentration expected in the sewer.

### 4.2 Overflow events

**Fig. 4:**
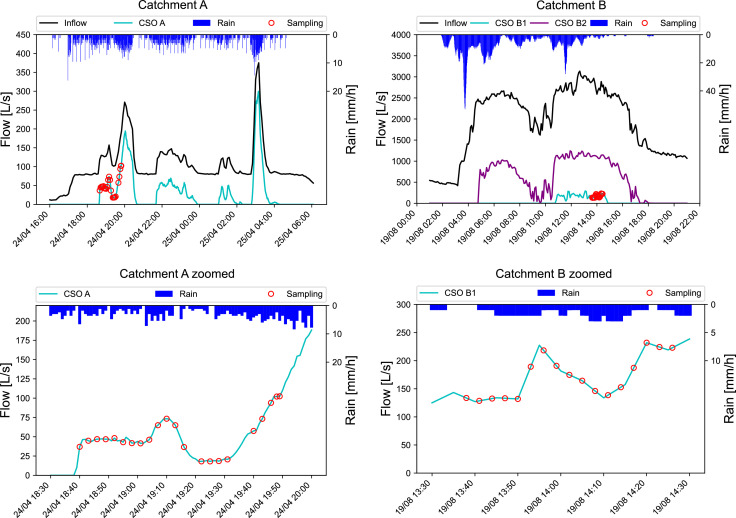
Inflow to CSOs, overflow discharge at CSOs, rain intensity, and sampling time points of the overflow event. Left: catchment A, right: catchment B, top: whole event, bottom: zoomed into sampling period.

An overview of the sampled overflow events at the two catchments can be found in Fig. 4.

**Catchment A.** The event took place on 24.04.2022 and 25.04.2022. Several overflows occurred between 18:30 and 04:30. The high-frequency samples were taken at the beginning of the overflow event from 18:30 to 19:50. During this time span, the overflow discharge varied between 50 and 100 L/s. The two additional events sampled at catchment A are depicted in SI Section 3.1.

**Catchment B.** The sampled event was on 19.08.2022 between 04:00 and 18:00. The high-frequency samples were taken from 13:35 to 14:25. The overflow discharge varied between 150 to 250 L/s.

### 4.3 Sampling

To investigate the high temporal fluctuations of organic micropollutants at CSOs, we collected samples from CSO A and CSO B1 at intervals of 3 min. We used an automated sampler (MAXX TP5C) with integrated cooling system and 24 glass bottles. The sample volume was 250 ml, which was taken by the automated sampler in less than 10 seconds.

To obtain information on the fluctuations within the 3 min, we also took 3 min composite samples. We continuously pumped wastewater from the same suction point into a 4 L beaker with a peristaltic pump at constant pumping rate (1.3 L/min). Every 3 min, we stirred the content of the beaker, took a sample, and emptied the beaker.

The samples were taken to the lab directly after sampling, where we aliquoted them (3 × 1 ml & 1 × 10 ml) and stored them in muffled glass vials in a freezer by –20 °C.

### 4.4 Analytical method for organic micropollutants

For sample analysis, Anliker et al.’s (2020) method was used with slight modifications. Briefly, each 1 ml sample was defrosted at ambient temperature before it was centrifuged (centrifugal force 3g, Heraeus Sepatech) to separate the solids from the liquid. From the supernatant, 800 µl was transferred to a glass vial and spike with 8 µL of a solution containing 50 µg/L isotope-labelled internal standard (see SI Table 1). For analysis, 100 µl of the sample was injected into a chromatographic column (Atlantis T3, C18, 5 µm, 3 mm × 15 cm) applying a water–methanol gradient (both containing 0.1 % formic acid, see SI 1.4). Analyte detection was performed on a high-resolution mass spectrometer (Q-Exactive, Thermo Fischer) after electrospray ionization (ESI) in two separate runs for positive and negative mode. Full-scan MS1 spectra at a resolution ® of 140,000 (at m/z 200) were acquired over the mass range m/z 100–1,000 followed by five data-dependent MS2 scans (R = 17,500 at m/z 200; triggered by target analyte masses). Every 10 samples, a blank, calibration point, and blind were measured for quality control. During the analysis, the samples were stored at 5 °C.

The calibration curve was mixed in Evian water and consisted of 11 concentration points between 5 ng/L and 10,000 ng/L for 53 substances with mostly matching internal standards. Selected samples were spiked with 250 ng/L and 2500 ng/L to calculate the relative recovery of the spiked analyte amounts in the matrix (see SI Section 1.3). Furthermore, aliquots’ measurements and multiple injections of the same aliquot were performed to determine the precision of the method (see SI Section 1.1). Stability data were used to check the stability of our target compounds over the sampling and freezing steps (see SI Section 1.2).

### 4.5 Additional measurements

**Catchment A.** In addition, we measured the rain intensity, overflow discharge into the receiving water body, and water level before the overflow crest. The overflow discharge was measured in a pipe with a diameter of 1 m with a Flo-Dar sensor. The water level was measured with a radar level sensor (micropilot FMR20, Endress & Hauser).

**Catchment B.** The WWTP operator provided us with rain data for the catchment and discharge measurements for inflow to the CSO and discharge of CSO B1 and B2 to the receiving water body.

### 4.6 Statistical analysis of temporal variation

#### 4.6.1 Quantitative description of fluctuation

To describe the observed concentration fluctuation quantitatively, we calculated the flashiness index (Eq. 1). The flashiness index is often used in hydrology to describe the oscillation of a temporal profile, typically the flow in a river, relative to the sum over the whole observation period (Baker et al., 2004).

(1) $$\begin{array}{ccc} & & \text{Flashiness}\;\text{index}=\sum \left|x_{i}-x_{i-1}\right|/\sum x_{i}\\ & & \;x_{i}:\;\text{value}\;\text{of}\;\text{element}\;\text{‘i'}\;\text{in}\;\text{temporal}\;\text{profile}\; \end{array}$$

We applied this formula to our measured concentration profiles, where x_i_ is the concentration of a micropollutant at one time point (every 3 min) of the 1 h events.

#### 4.6.2 Testing different sampling strategies

From the 3 min composite samples, we tested time proportional sampling strategies with intervals of 6, 9, 15, 30 and 60 min for all substances. First, the measured EMC was calculated from the 3 min composite sample according to Eq. 2.

(2) $$\begin{array}{ccc} & & \text{EMC}=\sum \left(c_{i}*V_{i}\right)/\sum V_{i}\\ & & \;c_{i}:\;\text{measured}\;\text{concentration}\;\text{of}\;\text{sample}\;\text{number}\;\text{‘i'}\\ & & \;V_{i}:\;\text{measured}\;\text{volume}\;\text{of}\;\text{stormwater}\;\text{of}\;\text{sample}\;\text{‘i'}\; \end{array}$$

 

**Fig. 5:**
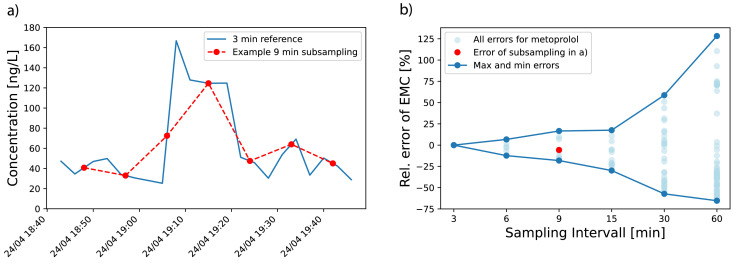
a) Time series of pharmaceutical metoprolol measured with 3 min composite samples (blue) and one from nine realisations of subsampling with 9 min interval (red). b) Relative errors of derived event mean concentration (EMC) when applying different sampling intervals with outer boundary of all possible errors (dark blue line) and error of realisation from a) (red dot).

From the 3 min composite samples, we took hypothetical samples for the chosen intervals and calculated the EMC of the selection by using the measured concentration and cumulated discharge volume during this interval (Fig. 5a). Next, we calculated the relative difference from the measured and the estimated EMC via Eq. 3. The outer boundaries of the possible outcomes are reported (Fig. 5b). This was done for all substances.

(3) $$\begin{array}{ccc} & & \text{Rel.}\;\text{error}=\left({\mathrm{EMC}}_{\mathrm{estimated}}-{\text{EMC}}_{\mathrm{measured}}\right)/{\text{EMC}}_{\mathrm{measured}}\times 100\;\left[\%\right]\\ & & \;{\text{EMC}}_{\text{estimated}}:\;\text{EMC}\;\text{estimated}\;\text{from}\;\text{intervals}\;>3\text{min}\\ & & \;{\text{EMC}}_{\text{measured}}:\;\text{EMC}\;\text{derived}\;\text{from}\;3\;\text{min}\;\text{composite}\;\text{samples} \end{array}$$

 

An alternative approach to determining the necessary sampling interval could be to analyse the frequency spectrum of the micropollutant concentrations. Such an approach would be interesting for determining the optimal sampling interval for evaluating the detailed concentration pattern over time for future work. However, in this study, we are focusing on the EMC assessment rather than the detailed concentration pattern over time.

#### 4.6.3 Difference between grab and composite samples

To check whether the frequency could be even higher than 3 min, we calculated the difference between the grab and composite samples (Eq. 4). We estimated the absolute difference between the two samples for each timepoint, added them up over the 1-hour event and divided by the number of points and the mean concentration of the grab samples.

(4) $$\begin{array}{ccc} & & {\text{Difference}}_{\mathrm{grab}\_\text{mix}}=\sum \left|c_{\text{mix,t}}-c_{\text{grab,t}}\right|/N/\text{EMC}\\ & & \;c_{\text{mix},t}:\;\text{measured}\;\text{concentration}\;\text{of}\;\text{mixed}\;\text{sample}\;\text{at}\;\text{time}\;t\\ & & \;c_{\text{grab},t}:\;\text{measured}\;\text{concentration}\;\text{of}\;\text{grab}\;\text{sample}\;\text{at}\;\text{time}\;t\\ & & \;N:\;\text{total}\;\text{number}\;\text{of}\;\text{samples}\\ & & \;\text{EMC}:\;\text{Event}\;\text{mean}\;\text{concentration} \end{array}$$

## Funding

This work was financially supported by the Swiss Federal Office for the Environment (FOEN) [grant nr. 19.0071.PJ / 8971CB0BA].

## Declaration of generative AI and AI-assisted technologies in the writing process

During the preparation of this work, the authors used ChatGPT in order to improve language. After using this tool/service, the authors reviewed and edited the content as needed and take full responsibility for the content of the publication.

## Declaration of Competing Interest

The authors declare the following financial interests/personal relationships which may be considered as potential competing interests: Viviane Furrer reports financial support was provided by Swiss Federal Office for the Environment (FOEN).

## Data Availability

All data is available through Eawag's Research Data Institutional Collection (ERIC-open) at https://doi.org/10.25678/0009A2.

## Appendix Supplementary materials

Image, application 1

## References

[bib0001] Anliker S., Loos M., Comte R., Ruff M., Fenner K., Singer H. (2020). Assessing emissions from pharmaceutical manufacturing based on temporal high-resolution mass spectrometry data. Environ. Sci. Technol..

[bib0002] Baker D.B., Richards R.P., Loftus T.T., Kramer J.W. (2004). A new flashiness index: characteristics and applications to midwestern rivers and streams. J. Am. Water Resour. Assoc..

[bib0003] Bollmann U.E., Vollertsen J., Carmeliet J., Bester K. (2014). Dynamics of biocide emissions from buildings in a suburban stormwater catchment - concentrations, mass loads and emission processes. Water Res..

[bib0004] Burkhardt M., Zuleeg S., Vonbank R., Schmid P., Hean S., Lamani X., Bester K., Boller M. (2011). Leaching of additives from construction materials to urban storm water runoff. Water Sci. Technol..

[bib0005] Gasperi J., Zgheib S., Cladiere M., Rocher V., Moilleron R., Chebbo G. (2012). Priority pollutants in urban stormwater: part 2 - case of combined sewers. Water Res..

[bib0006] Launay M.A., Dittmer U., Steinmetz H. (2016). Organic micropollutants discharged by combined sewer overflows - characterisation of pollutant sources and stormwater-related processes. Water Res..

[bib0007] Madoux-Humery A.S., Dorner S., Sauve S., Aboulfadl K., Galarneau M., Servais P., Prevost M. (2013). Temporal variability of combined sewer overflow contaminants: evaluation of wastewater micropollutants as tracers of fecal contamination. Water Res..

[bib0008] McCarthy D.T., Zhang K., Westerlund C., Viklander M., Bertrand-Krajewski J.L., Fletcher T.D., Deletic A. (2018). Assessment of sampling strategies for estimation of site mean concentrations of stormwater pollutants. Water Res..

[bib0009] Musolff A., Leschik S., Reinstorf F., Strauch G., Schirmer M. (2010). Micropollutant Loads in the Urban Water Cycle. Environ. Sci. Technol..

[bib0010] Mutzner L., Furrer V., Castebrunet H., Dittmer U., Fuchs S., Gernjak W., Gromaire M.C., Matzinger A., Mikkelsen P.S., Selbig W.R., Vezzaro L. (2022). A decade of monitoring micropollutants in urban wet-weather flows: what did we learn?. Water Res..

[bib0011] Mutzner L., Staufer P., Ort C. (2016). Model-based screening for critical wet-weather discharges related to micropollutants from urban areas. Water Res..

[bib0012] Nickel J.P., Sacher F., Fuchs S. (2021). Up-to-date monitoring data of wastewater and stormwater quality in Germany. Water Res..

[bib0013] Ort C., Lawrence M., Reungoat J., Mueller J.F. (2010).

[bib0014] Ort C., Lawrence M., Rieckermann J., Joss A. (2010).

[bib0015] Peter K.T., Hou F., Tian Z., Wu C., Goehring M., Liu F., Kolodziej E.P. (2020). More than a first flush: urban creek storm hydrographs demonstrate broad contaminant pollutographs. Environ. Sci. Technol..

[bib0016] Petrie B. (2021). A review of combined sewer overflows as a source of wastewater-derived emerging contaminants in the environment and their management. Environ. Sci. Pollut. Res. Int..

[bib0017] Phillips P., Chalmers A., Gray J., Kolpin D.W., Foreman W.T., Wall G.R. (2012). Combined sewer overflows: an environmental source of hormones and wastewater micropollutants. Environ. Sci. Technol..

[bib0018] Pouzol T., Levi Y., Bertrand-Krajewski J.L. (2020). Modelling daily and hourly loads of pharmaceuticals in urban wastewater. Int. J. Hyg. Environ. Health.

[bib0019] Rieckermann J. (2005). Dispersion coefficients of sewers from tracer experiments. Water Sci. Technol..

[bib0020] Spahr S., Teixidó M., Sedlak D.L., Luthy R.G. (2020). Hydrophilic trace organic contaminants in urban stormwater: occurrence, toxicological relevance, and the need to enhance green stormwater infrastructure. Environ. Sci.: Water Res. Technol..

[bib0021] Tolouei S., Burnet J.B., Autixier L., Taghipour M., Bonsteel J., Duy S.V., Sauve S., Prevost M., Dorner S. (2019). Temporal variability of parasites, bacterial indicators, and wastewater micropollutants in a water resource recovery facility under various weather conditions. Water Res..

[bib0022] Wittmer I.K., Bader H.P., Scheidegger R., Singer H., Luck A., Hanke I., Carlsson C., Stamm C. (2010). Significance of urban and agricultural land use for biocide and pesticide dynamics in surface waters. Water Res..

