# Supplementary material for: Micropollutant concentration fluctuations in combined sewer overflows require short sampling intervals
Source: Water Res X. 2023 Sep 12;21:100202. doi: 10.1016/j.wroa.2023.100202 (PMC10719572; doi:10.1016/j.wroa.2023.100202)
Supplement: Supplementary file 1 [file mmc1.docx]

##

## Supporting Information

Micropollutant concentration fluctuations in combined sewer overflows require short sampling intervals

Viviane Furrer^1,2^, Lena Mutzner^1^, Christoph Ort^1^, Heinz Singer^1^

1 Eawag, Swiss Federal Institute of Aquatic Science and Technology, 8600 Dübendorf, Switzerland.

2 Institute of Civil, Environmental and Geomatic Engineering, ETH Zürich, 8093 Zurich, Switzerland.

*Corresponding author: christoph.ort@eawag.ch

# Target substances

Table 1: List with all analysed substances, their InChiKey for identification, the observed level of quantification (LOQ) and used isotope labelled compound for quantification. PPP = Plant protection product, Sweet = Artificial sweetener.

| **Groupe** | **Substance** | **Use** | **InChiKey** | **LOQ [ng/L]** | **Isotope labelled compound** |
| --- | --- | --- | --- | --- | --- |
| **Indoor** | Acesulfame | Sweet | YGCFIWIQZPHFLU-UHFFFAOYSA-N | 50 | Acesulfame-D4 |
|  | Azithromycin | Pharma | MQTOSJVFKKJCRP-BICOPXKESA-N | 100 | Azithromycin-D3 |
|  | Atenolol | Pharma | METKIMKYRPQLGS-UHFFFAOYSA-N | 10 | Atenolol-D7 |
|  | Bezafibrate | Pharma | IIBYAHWJQTYFKB-UHFFFAOYSA-N | 10 | Bezafibrate-D4 |
|  | Candesartan | Pharma | HTQMVQVXFRQIKW-UHFFFAOYSA-N | 10 | Candesartan-D5 |
|  | Carbamazepine | Pharma | FFGPTBGBLSHEPO-UHFFFAOYSA-N | 5 | Carbamazepine-D8 |
|  | Citalopram | Pharma | WSEQXVZVJXJVFP-UHFFFAOYSA-N | 5 | Citalopram-D6 |
|  | Clarithromycin | Pharma | AGOYDEPGAOXOCK-KCBOHYOISA-N | 10 | Clarithromycin-D3 |
|  | Caffeine | Lifestyle | RYYVLZVUVIJVGH-UHFFFAOYSA-N | 100 | Caffeine-D9 |
|  | Cyclamate | Sweet | HCAJEUSONLESMK-UHFFFAOYSA-N | 50 | Cyclamate-D11 |
|  | Diclofenac | Pharma | DCOPUUMXTXDBNB-UHFFFAOYSA-N | 10 | Diclofenac-D4 |
|  | Erythromycin | Pharma | ULGZDMOVFRHVEP-RWJQBGPGSA-N | 50 | Erythromycin-13C3-D3 |
|  | Gabapentin | Pharma | UGJMXCAKCUNAIE-UHFFFAOYSA-N | 50 | Gabapentin-D4 |
|  | Hydrochlorothiazide | Pharma | JZUFKLXOESDKRF-UHFFFAOYSA-N | 25 | Hydrochlorothiazide-C13-D2 |
|  | Ibuprofen | Pharma | HEFNNWSXXWATRW-UHFFFAOYSA-N | 250 | Ibuprofen-D3 |
|  | Iopamidol | Pharma | XQZXYNRDCRIARQ-UHFFFAOYSA-N | 250 | Iopamidol-D3 |
|  | Irbesartan | Pharma | YOSHYTLCDANDAN-UHFFFAOYSA-N | 25 | Irbesartan-D3 |
|  | Lamotrigine | Pharma | PYZRQGJRPPTADH-UHFFFAOYSA-N | 5 | Lamotrigine-13C3-D3 |
|  | Levetiracetam | Pharma | HPHUVLMMVZITSG-LURJTMIESA-N | 25 | Levetiracetam-D3 |
|  | Lidocaine | Pharma | NNJVILVZKWQKPM-UHFFFAOYSA-N | 10 | Lidocaine-D10 |
|  | Mefenamic acid | Pharma | HYYBABOKPJLUIN-UHFFFAOYSA-N | 10 | Mefenamic acid-D3 |
|  | Metformin | Pharma | XZWYZXLIPXDOLR-UHFFFAOYSA-N | 100 | Metformin-D6 |
|  | Metoprolol | Pharma | IUBSYMUCCVWXPE-UHFFFAOYSA-N | 25 | Metoprolol-D7 |
|  | Naproxen | Pharma | CMWTZPSULFXXJA-VIFPVBQESA-N | 100 | Naproxen-D3 |
|  | Paracetamol (3-Acetamidophenol) | Pharma | RZVAJINKPMORJF-UHFFFAOYSA-N | 50 | 3-Acetamidophenol-D4 |
|  | Pravastatin | Pharma | TUZYXOIXSAXUGO-PZAWKZKUSA-N |  | Pravastatin-D3 |
|  | Saccharin | Sweet | CVHZOJJKTDOEJC-UHFFFAOYSA-N | 50 | Saccharin-D4 |
|  | Sitagliptin | Pharma | MFFMDFFZMYYVKS-SECBINFHSA-N | 10 | Sitagliptin-D4 |
|  | Sulfamethoxazole | Pharma | JLKIGFTWXXRPMT-UHFFFAOYSA-N | 10 | Sulfamethoxazole-D4 |
|  | Tramadol | Pharma | TVYLLZQTGLZFBW-ZBFHGGJFSA-N | 10 | Tramadol-D6 |
|  | Triclosan | Biocide | XEFQLINVKFYRCS-UHFFFAOYSA-N | 10 | Triclosan-D3 |
|  | Trimethoprim | Pharma | IEDVJHCEMCRBQM-UHFFFAOYSA-N | 5 | Trimethoprim-D3 |
|  | Venlafaxine | Pharma | PNVNVHUZROJLTJ-UHFFFAOYSA-N | 10 | Venlafaxine-D6 |
| **Outdoor** | 2-n-Octyl-4-isothiazolin-3-on (OIT) | Biocide | JPMIIZHYYWMHDT-UHFFFAOYSA-N | 5 | Octhilinon-D17 |
|  | 2-4-D |  | OVSKIKFHRZPJSS-UHFFFAOYSA-N | 5 | 2-4-D-D3 |
|  | Atrazine | Legacy PPP | MXWJVTOOROXGIU-UHFFFAOYSA-N | 5 | Atrazine-D5 |
|  | Bentazone | PPP | ZOMSMJKLGFBRBS-UHFFFAOYSA-N | 10 | Bentazone-D6 |
|  | Boscalid | PPP | WYEMLYFITZORAB-UHFFFAOYSA-N | 10 | Boscalid-D4 |
|  | Carbendazim | Legacy PPP, biocide | TWFZGCMQGLPBSX-UHFFFAOYSA-N | 5 | Carbendazim-D4 |
|  | Dicamba | PPP | IWEDIXLBFLAXBO-UHFFFAOYSA-N | 250 | Dicamba-D3 |
|  | Diuron | Legacy PPP, biocide | XMTQQYYKAHVGBJ-UHFFFAOYSA-N | 10 | Diuron-D6 |
|  | Epoxiconazole | Legacy PPP | ZMYFCFLJBGAQRS-IAGOWNOFSA-N | 5 | Epoxiconazole-D4 |
|  | Imidacloprid | Legacy PPP, biocide | YWTYJOPNNQFBPC-UHFFFAOYSA-N | 10 | Imidacloprid-D4 |
|  | Isoproturon | Legacy PPP, biocide | PUIYMUZLKQOUOZ-UHFFFAOYSA-N | 5 | Isoproturon-D6 |
|  | MCPA | PPP | WHKUVVPPKQRRBV-UHFFFAOYSA-N | 10 | MCPA-D3 |
|  | Mecoprop | PPP | WNTGYJSOUMFZEP-UHFFFAOYSA-N | 10 | Mecoprop-D6 |
|  | Metolachlor | PPP | WVQBLGZPHOPPFO-UHFFFAOYSA-N | 10 | Metolachlor-D6 |
|  | Metribuzin | PPP | FOXFZRUHNHCZPX-UHFFFAOYSA-N |  | Metribuzin-(S-methyl-D3) |
|  | Terbutryn + Prometryn | PPP, biocide | IROINLKCQGIITA-UHFFFAOYSA-N | 10 | Terbutryn-D5 |
|  | Terbuthylazine | PPP, legacy biocide | FZXISNSWEXTPMF-UHFFFAOYSA-N |  | Terbuthylazine-D5 |
|  | Thiamethoxam | Legacy PPP, biocide | NWWZPOKUUAIXIW-UHFFFAOYSA-N |  | Thiamethoxam-D3 |
|  | 1-3-Diphenylguanidine | Road | OWRCNXZUPFZXOS-UHFFFAOYSA-N | 10 | Lidocain-D10 |
|  | 6PPD_quinone | Road | UBMGKRIXKUIXFQ-UHFFFAOYSA-N | 10 | 6PPD_quinone-D5 |
|  | Hexa(methoxymethyl)  melamine (HMMM) | Road | BNCADMBVWNPPIZ-UHFFFAOYSA-N | 25 | Terbutryn-D5 |
| **Several** | DEET (N-N-diethyl-3-methylbenzamide) | Biocide | MMOXZBCLCQITDF-UHFFFAOYSA-N | 10 | DEET-D10 |

## Precision

Table 2: Coefficient of variation of 5 replicates (5 times same sample analysed), of 5 aliquotes taken after defrosting, and of 5 aliquotes taken in the field from the same automated sampler bottle.

| **Compound** | **Coefficient of Variation [%]** | | |  |
| --- | --- | --- | --- | --- |
|  | **Replicates** | **Aliquote Lab** | **Aliquote Field** | |
| 1-3-Diphenylguanidine | 2 | 4 | 3 | |
| 6PPD-quinone | 4 | 4 | 5 | |
| Atenolol | 3 | 4 | 4 | |
| Candesartan | 5 | 3 | 6 | |
| Carbamazepine | 21 | 5 | 4 | |
| Citalopram | 7 | 2 | 5 | |
| Clarithromycin | 14 | 13 | <LOQ | |
| Caffeine | 4 | 3 | 5 | |
| Diclofenac | 1 | 3 | 7 | |
| Diuron | 3 | 3 | 5 | |
| Gabapentin | 5 | 7 | 5 | |
| HMMM | 3 | 2 | 4 | |
| Ibuprofen | 13 | 3 | 11 | |
| Irbesartan | 5 | 3 | 6 | |
| Lamotrigine | 4 | 2 | 4 | |
| Levetiracetam | 7 | 6 | 8 | |
| Lidocaine | 3 | 6 | 5 | |
| Mefenamic acid | 2 | 4 | 4 | |
| Metoprolol | 3 | 5 | 5 | |
| OIT | 5 | 16 | 5 | |
| Sitagliptin | 3 | 5 | 4 | |
| Sulfamethoxazole | 3 | 9 | 8 | |
| Tramadol | 7 | 3 | 4 | |
| Trimethoprim | 5 | 7 | 7 | |
| Venlafaxine | 5 | 4 | 5 | |

## Stability

The samples used for this study were stored at 4°C in the automated sampler for maximum 3 days (normally only 24 h) and afterwards at -20°C for one to 6 months. No significant substance losses were observed for the here analysed substances for this storage time.


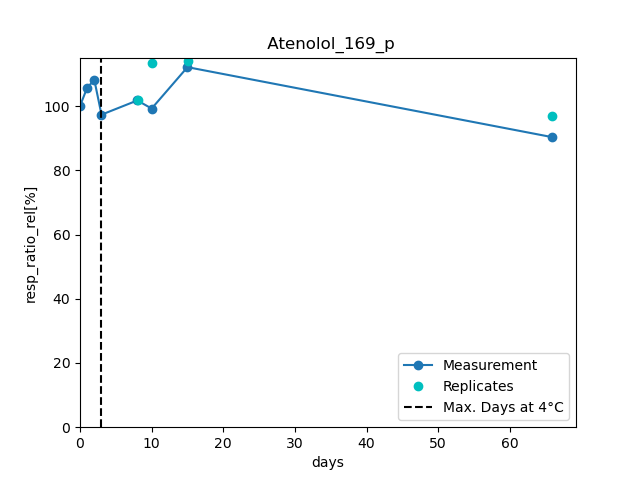

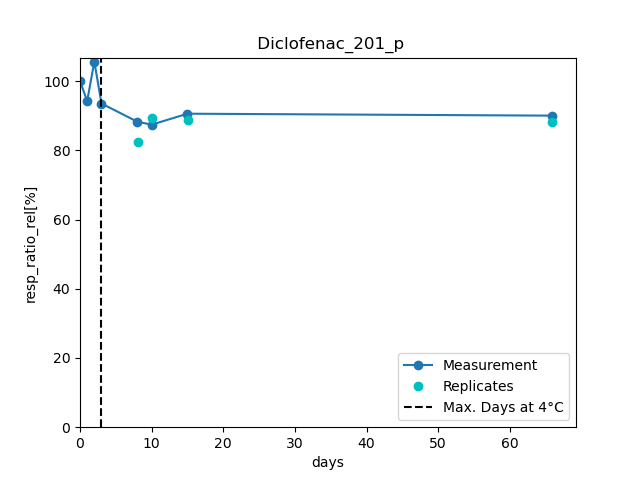


Figure 1: Decay curve of two example substances Atenolol and Diclofenac.

## Relative recovery

To quantify the relative recovery, 4 samples were spiked with a known concentration (2x 250 ng/L, 2x 2’500 ng/L). The relative recovery was calculated with formula 1.

Relative recovery [%] = (concenctration_spiked_sample - concentration_unspiked_sample) / spiked_concentration * 100 (1)

Table 4: Relative recovery from 4 spiked samples and their unspiked concentrations.

| **Compound** | **Concentration in samples [ng/L]** | | | | **Rel recovery [%]** | | | |
| --- | --- | --- | --- | --- | --- | --- | --- | --- |
|  | **S1** | **S2** | **S3** | **S4** | **S1 spiked 250** | **S2 spiked 250** | **S3 spiked 2500** | **S4 spiked 2500** |
| 1-3-Diphenylguanidine | 821 | 414 | 753 | 387 | 83 | 99 | 93 | 101 |
| 2-4-D | <LOQ | 11 | 9 | 11 | 106 | 99 |  |  |
| OIT | <LOQ | 12 | <LOQ | 9 | 113 | 103 |  |  |
| 6PPD_quinone | 34 | 7 | 35 | 9 | 154 | 135 |  |  |
| Acesulfame | 1390 | 3683 | 1793 | 3089 |  |  | 95 | 103 |
| Atenolol | 9 | 102 | <LOQ | 423 | 101 | 104 |  |  |
| Candesartan | 37 | 17 | 59 | 33 | 105 | 100 |  |  |
| Carbamazepine | <LOQ | 17 | <LOQ | 6 | 117 | 113 |  |  |
| Citalopram | <LOQ | 8 | 5 | 11 | 100 | 98 |  |  |
| Clarithromycin | <LOQ | 13 | 123 | 11 | 99 | 87 |  |  |
| Caffeine | 4434 | 8211 | 4507 | 9658 |  |  | 142 | 124 |
| Cyclamate | 3258 | 4824 | 6730 | 13468 |  |  | 81 | 78 |
| Diclofenac | 343 | 3150 | 399 | 1458 |  |  | 102 | 99 |
| Diuron | 8 | 15 | 8 | 14 | 111 | 107 |  |  |
| Gabapentin | 48 | 736 | 21 | 213 | 92 | 86 |  |  |
| HMMM | 446 | 569 | 419 | 543 | 105 | 105 | 103 | 110 |
| Hydrochlorothiazide | 78 | 258 | 67 | 303 | 100 | 94 |  |  |
| Ibuprofen_Na | 138 | 289 | 170 | 206 | 123 | 119 |  |  |
| Irbesartan | 189 | 18 | 67 | 12 | 96 | 83 |  |  |
| Isoproturon | <LOQ | <LOQ | <LOQ | <LOQ | 101 | 102 |  |  |
| Lamotrigine | 11 | 15 | 10 | 15 | 101 | 104 |  |  |
| Levetiracetam | 185 | 182 | 65 | 194 | 102 | 101 |  |  |
| Lidocaine | 209 | 14 | 40 | 16 | 105 | 104 |  |  |
| MCPA | <LOQ | 10 | 7 | 11 | 101 | 95 |  |  |
| Mecoprop | 123 | 182 | 138 | 185 | 106 | 110 |  |  |
| Mefenamic acid | 24 | 234 | 222 | 84 | 108 | 113 |  |  |
| Metformin | 3424 | 3655 | 4409 | 2746 |  |  | 112 | 130 |
| Metolachlor | <LOQ | <LOQ | <LOQ | <LOQ | 109 | 102 |  |  |
| Metoprolol | 16 | 17 | 56 | 19 | 100 | 100 |  |  |
| Metribuzin | <LOQ | <LOQ | <LOQ | <LOQ |  |  |  |  |
| DEET | 18 | 37 | 21 | 27 | 115 | 108 |  |  |
| Naproxen | 145 | 75 | 132 | 68 | 87 | 96 |  |  |
| Paracetamol | 2427 | 10856 | 673 | 10278 |  |  | 103 | 159 |
| Sitagliptin | 33 | 45 | 67 | 43 | 98 | 93 |  |  |
| Sulfamethoxazole | <LOQ | <LOQ | <LOQ | <LOQ | 103 | 96 |  |  |
| Tramadol | <LOQ | <LOQ | <LOQ | <LOQ | 110 | 102 |  |  |
| Trimethoprim | <LOQ | <LOQ | <LOQ | <LOQ | 104 | 104 |  |  |
| Venlafaxine | 13 | 36 | 8 | 45 | 106 | 101 |  |  |

## Analytical method

The analytical method was the same as described in Anliker et al. (2020), however with a slightly different water-methanol gradient. We had a 33 min chromatographic run that started with 0% methanol (channel B) for the first 1.5 min, then increased linearly to 95% methanol in 17 min, stayed at this condition for 10 min and finally decreased to 0% methanol in 0.5 min for 4 min re-equilibration (Figure 1). Source and MS settings were identical as described in table SI S4 of Anliker et al. (2020).


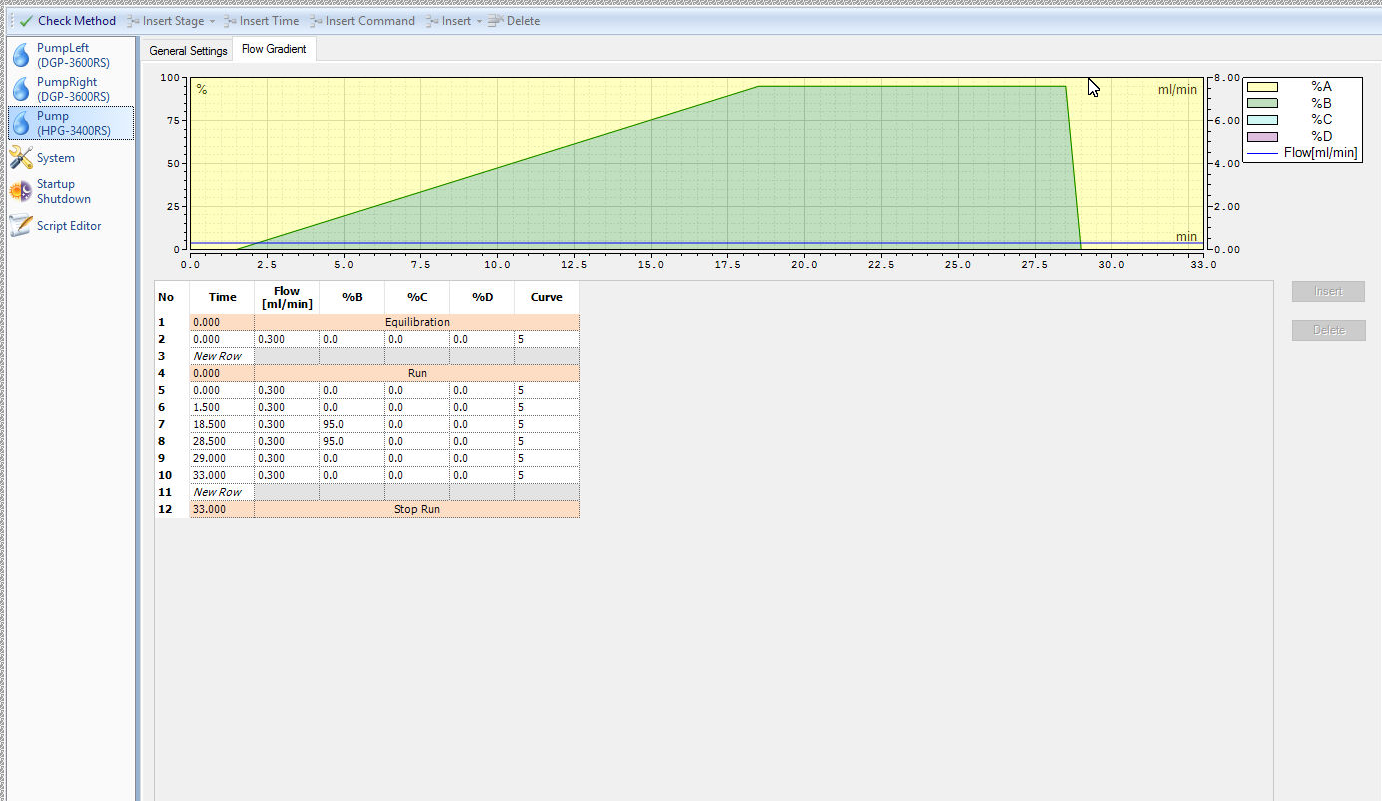


Figure 2: Water-methanol gradient of chromatographic run. Channel A: water, channel B: methanol.

# Concentrations of all evaluated target substances

Table 5: All substances with no or only few detections above LOQ.

| **Substance** | **LOQ** |
| --- | --- |
| Azithromycin | < 250 ng/L |
| Erythromycin | < 100 ng/L |
| Terbutryn + Prometryn | < 10 ng/L |
| Atrazine | < 5 ng/L |
| Bentazone | < 10 ng/L |
| Boscalid | < 25 ng/L |
| Dicamba | < 250 ng/L |
| Epoxiconazole | < 10 ng/L |
| Metolachlor | < 10 ng/L |
| Metribuzin | < 5 ng/L |
| Terbuthylazine | < 5 ng/L |
| Iopamidol | < 250 ng/L |
|  |  |

##
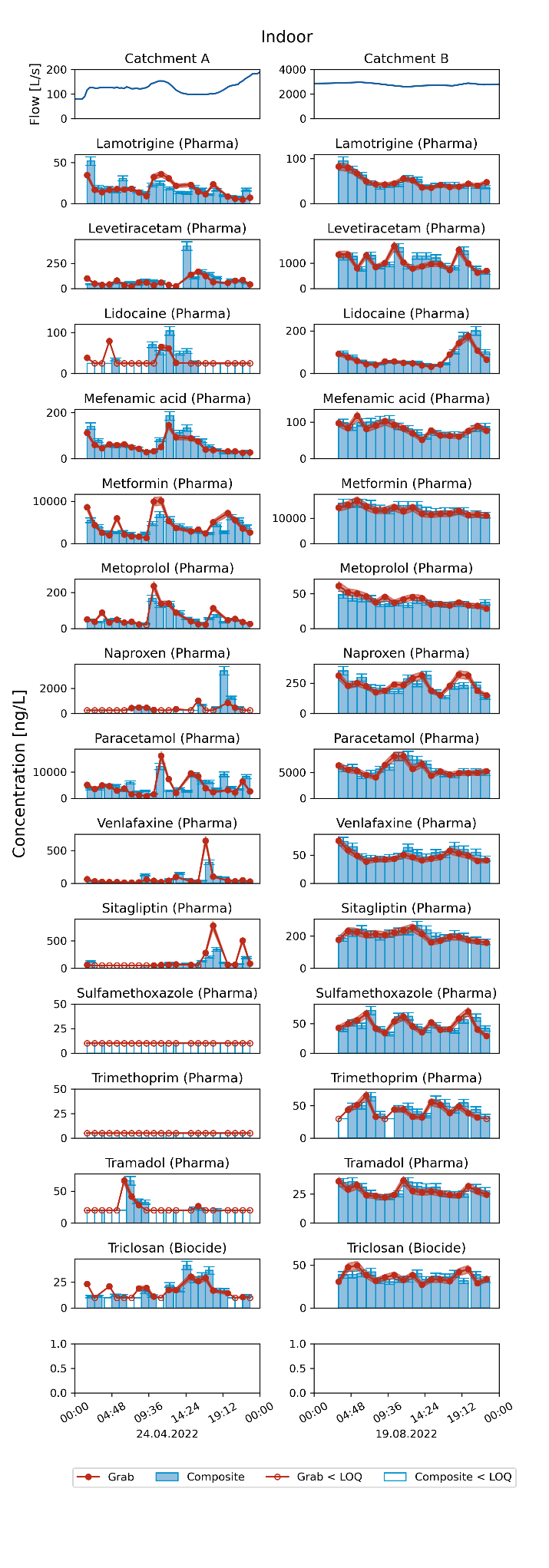
Additional substances in catchment A (event 24.4.2022) and catchment B


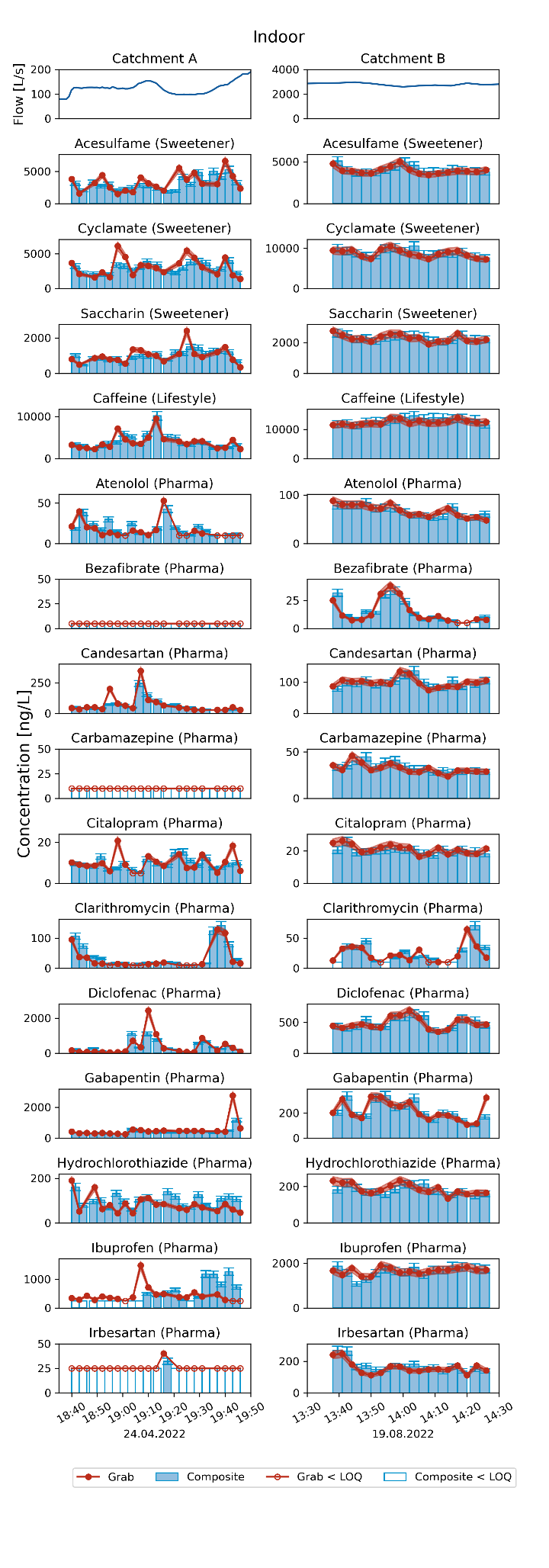


Figure 5: Flow (inflow to CSO) and concentration (with 10% error band) of indoor substances from 3-minute grab (red dots) and 3-minute continuous composite samples (blue bars) of overflow event from 24.4.2022 in catchment A and event from 19.8.2022 in catchment B.


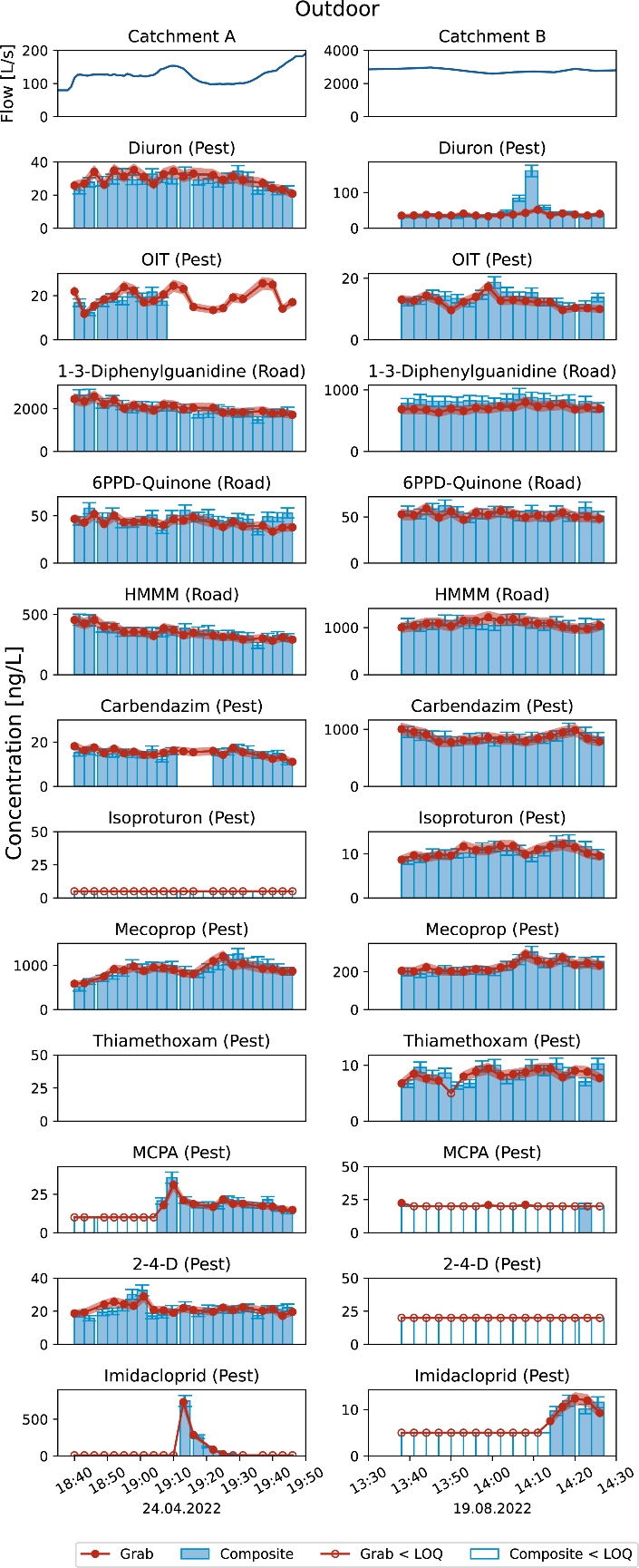


Figure 6: Flow (inflow to CSO) and concentration (with 10% error band) of outdoor substances from 3-minute grab (red dots) and 3-minute continuous composite samples (blue bars) of overflow event from 24.4.2022 in catchment A and event from 19.8.2022 in catchment B.

## MP concentrations of additional events in catchment A


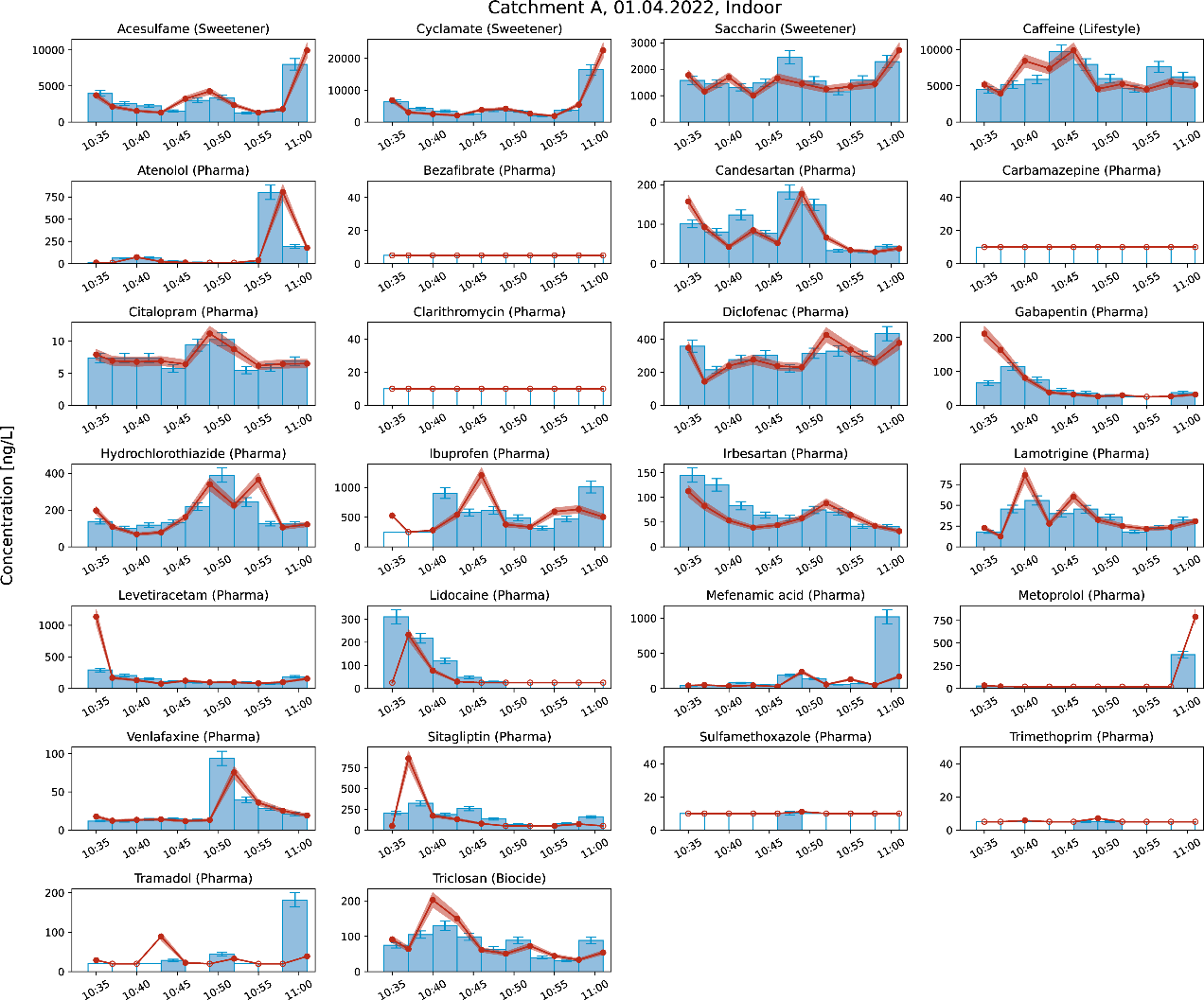


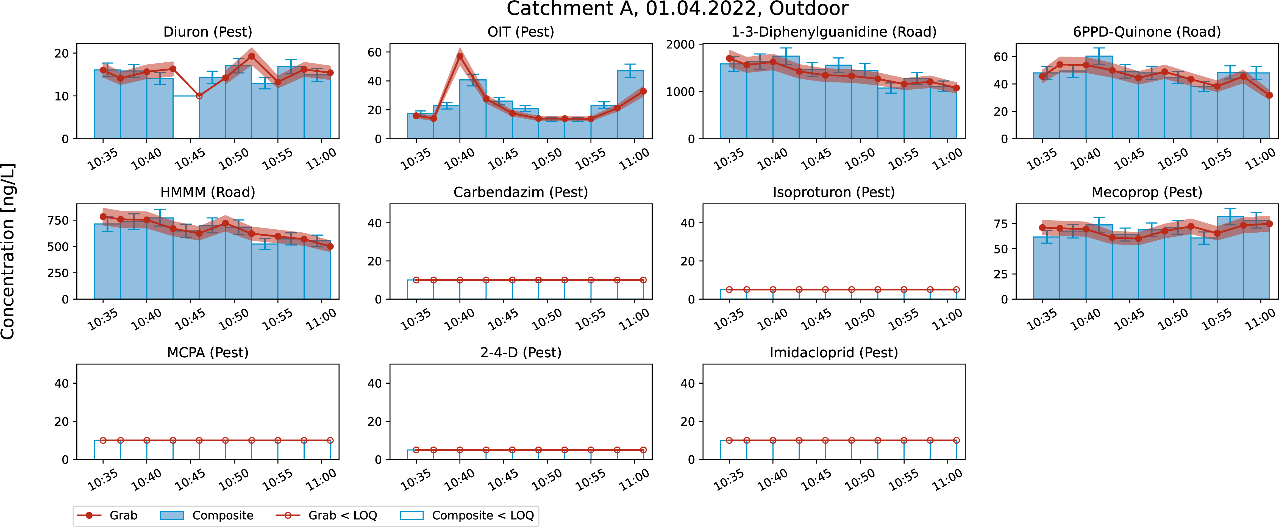


Figure 8: Concentration (with 10% error band) of indoor and outdoor substances from 3-minute grab (red dots) and 3-minute continuous composite samples (blue bars) of overflow event from 01.04.2022 in catchment A.


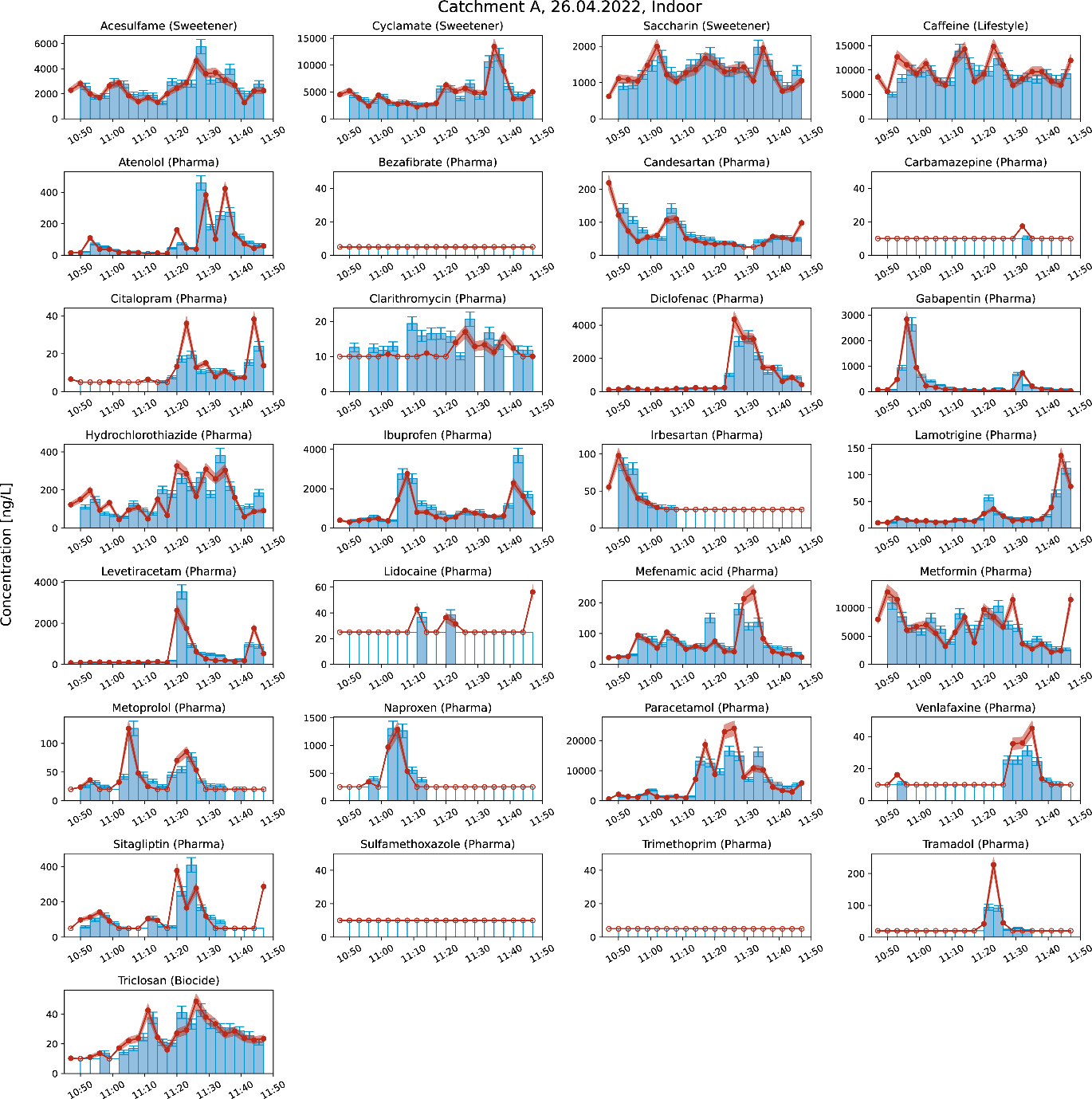


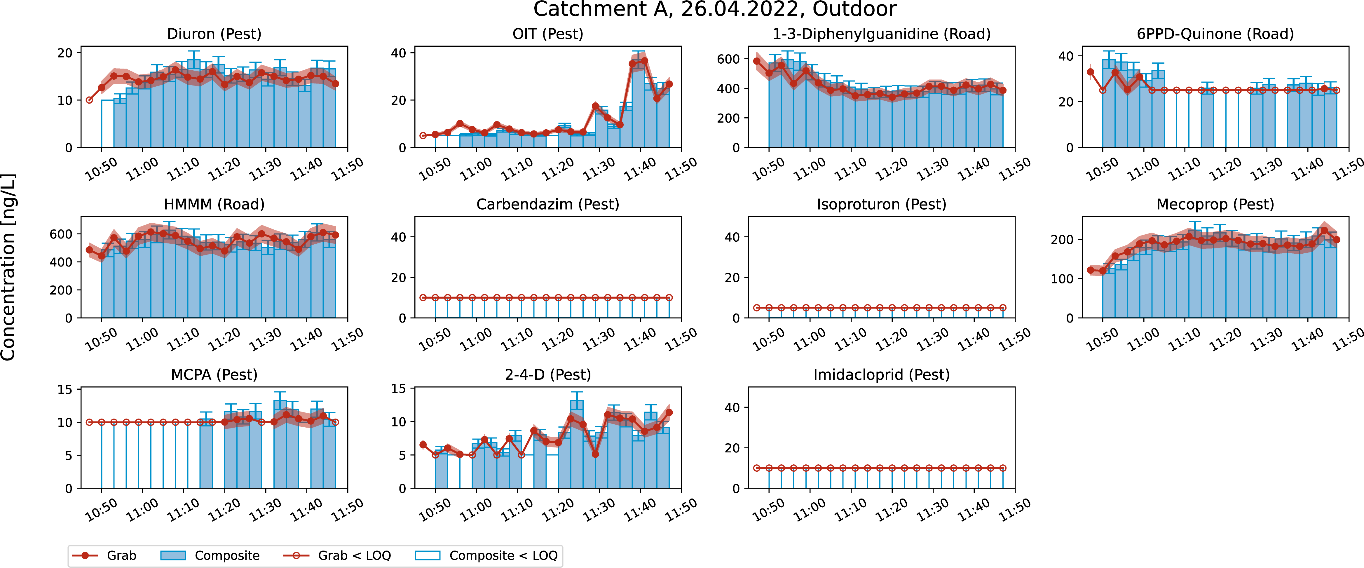


Figure 10: Concentration (with 10% error band) of indoor and outdoor substances from 3-minute grab (red dots) and 3-minute continuous composite samples (blue bars) of overflow event from 26.04.2022 in catchment A.

# Additional events at CSO in catchment A

## Overflow events


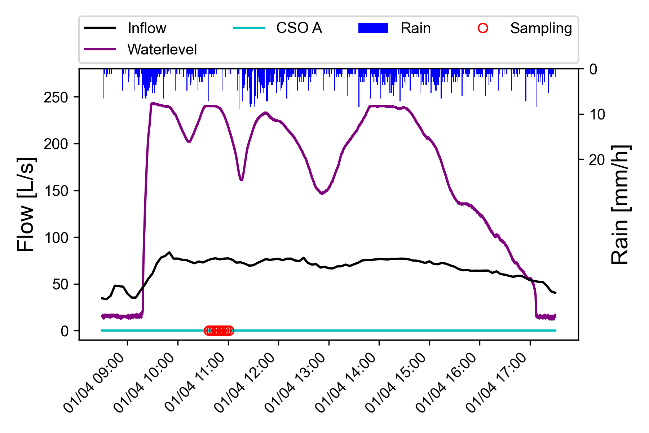

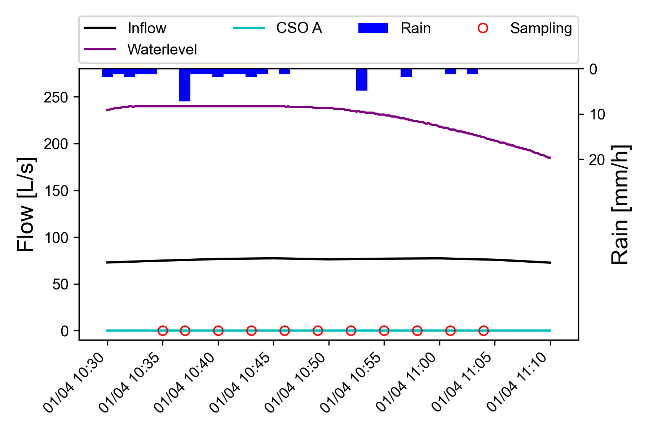


Figure 3: Inflow to CSO A, overflow discharge at CSO A, rain intensity, and sampling time points of the overflow event from 1.4.2022. Left: whole event, right: zoomed into sampling period.


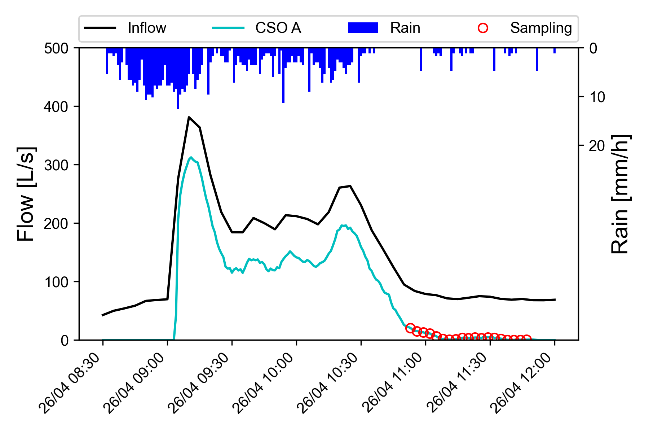

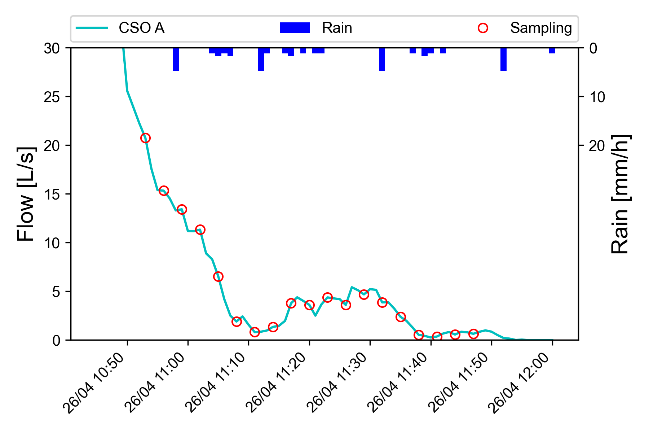


Figure 4: Inflow to CSO A, overflow discharge at CSO A, rain intensity, and sampling time points of the overflow event from 26.4.2022. Left: whole event, right: zoomed into sampling period.

## Comparison of dynamics of the three events at catchment A


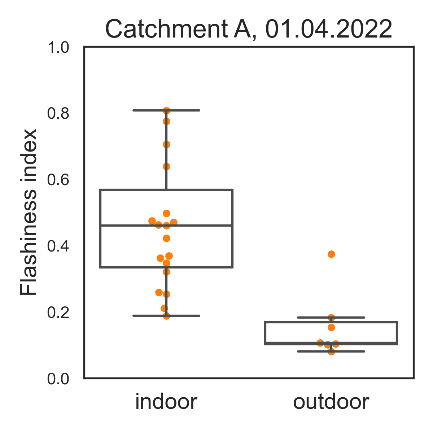

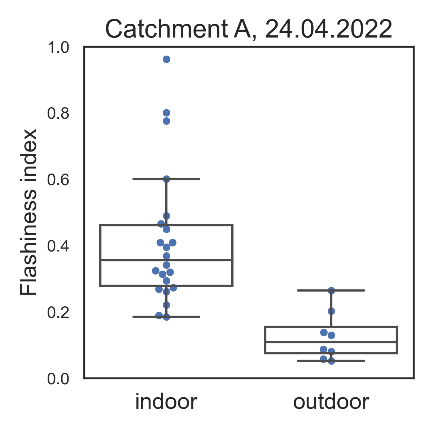

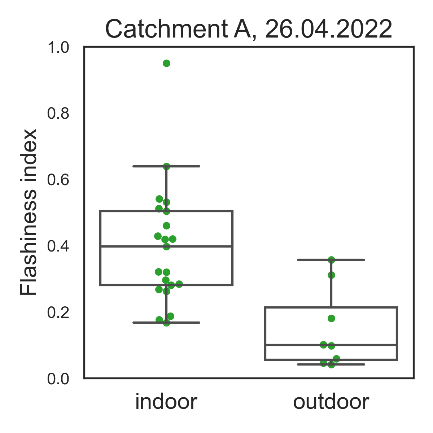


Figure 11: Boxplot of flashiness index of 3-minute composite samples at catchment A for all substances, distinguished between indoor and outdoor application. Left: event from 1.4.2022, middle: event from 24.4.2022, right: event from 26.4.2022.

# Difference of grab and composite samples


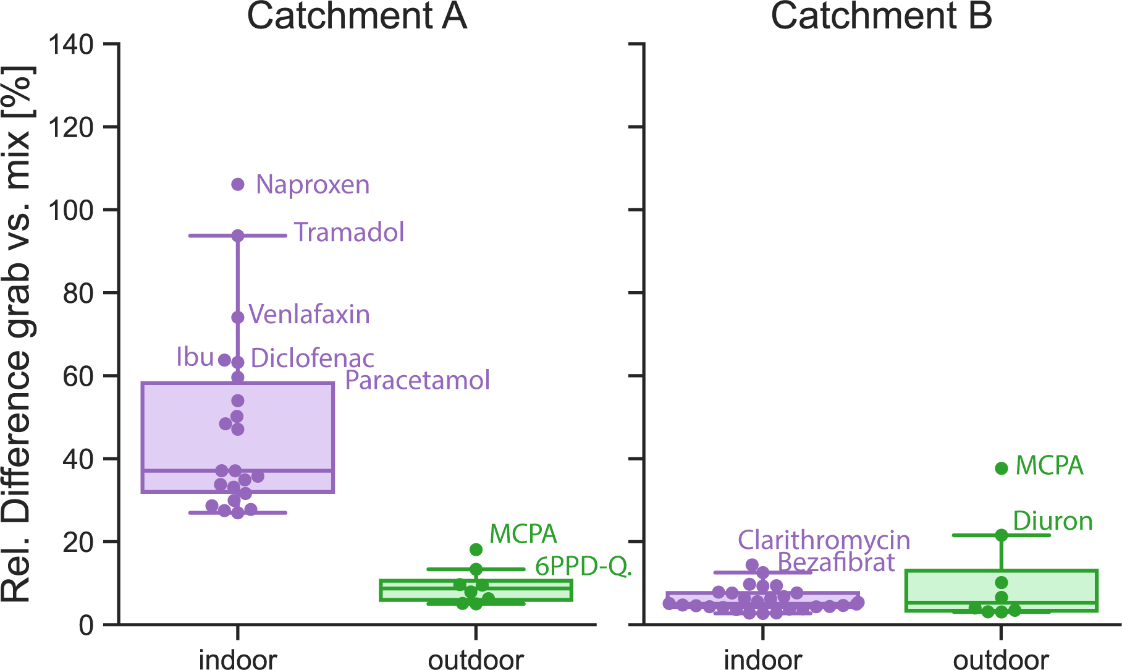


Figure 13: Boxplot of relative differences of 3-minute grab and composite samples for all substances, distinguished between indoor and outdoor application. Left: catchment A. Right: catchment B.

# Testing different sampling strategies


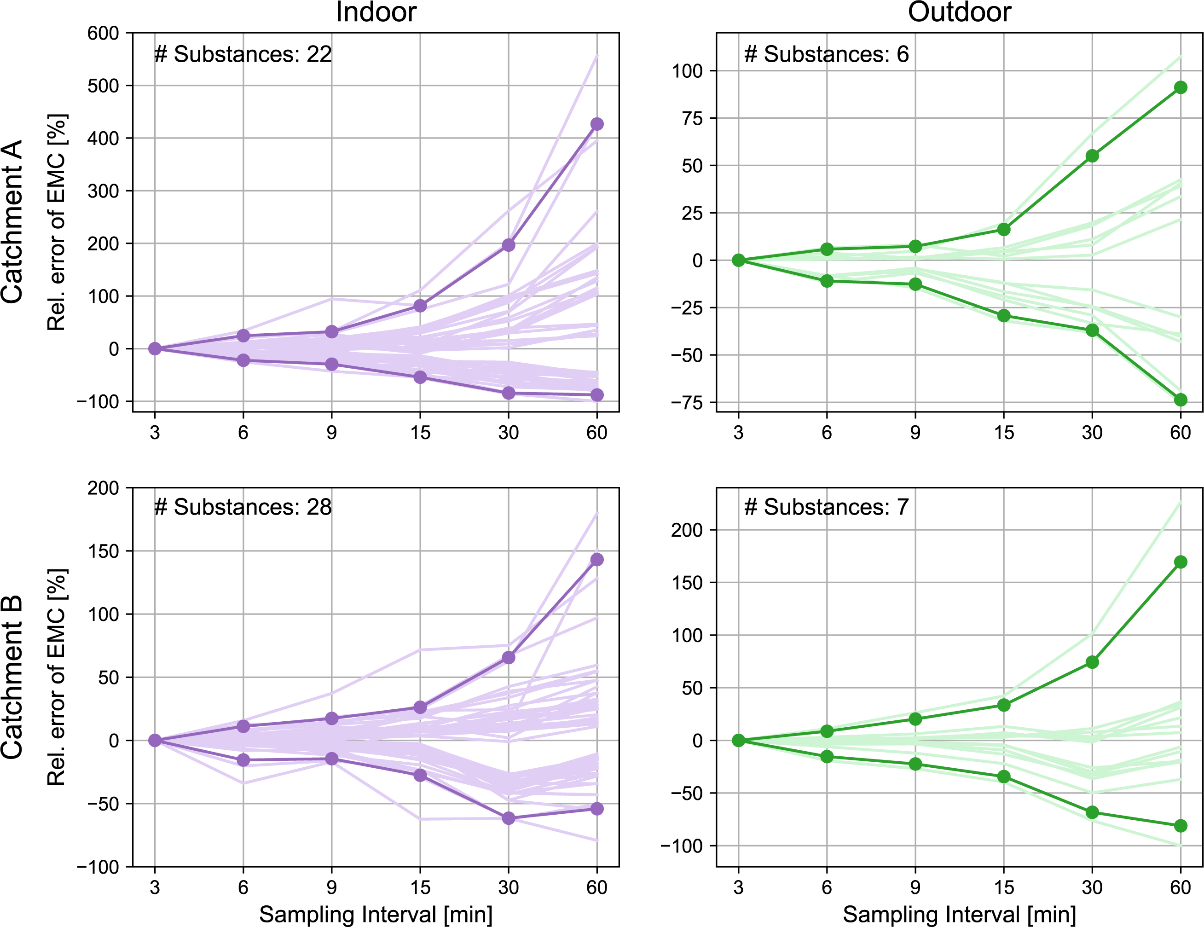


Figure 12: Maximum relative error of event mean concentration for each substance (light-coloured lines) and 95% quantile across all substances (dark lines) for various sampling intervals for indoor (left) and outdoor (right) substances, for catchment A (top, 2,700 P, event from 24.04.2022) and catchment B (bottom, 159,000 P, event from 19.08.2022) with zoomed y-axis.

# Study sites

## Catchment A


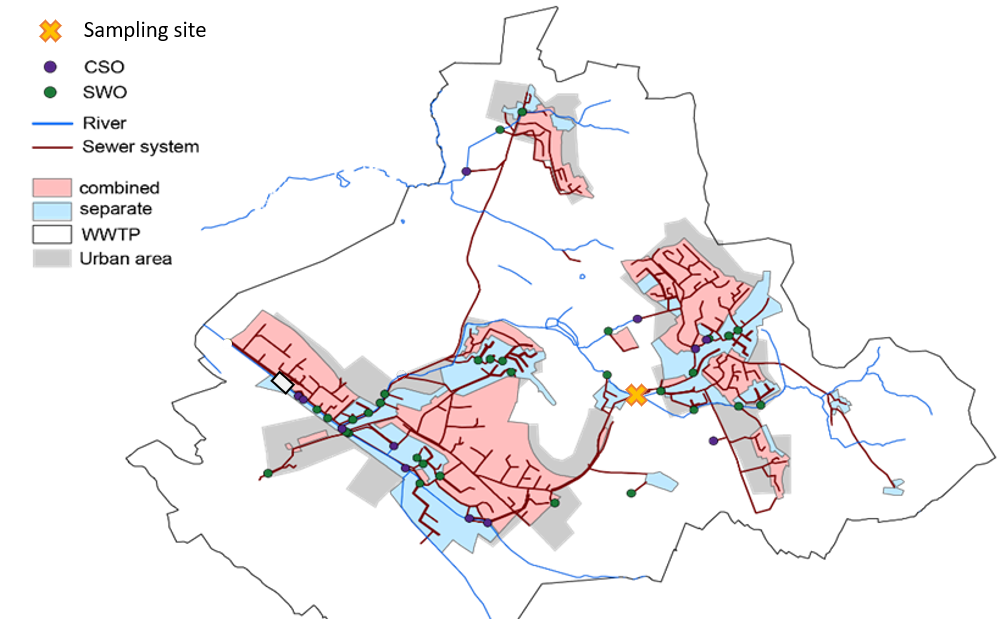


Figure 14: Scheme of catchment A with sewer system, urban area and sampling site (graphic adapted from Lena Mutzner).


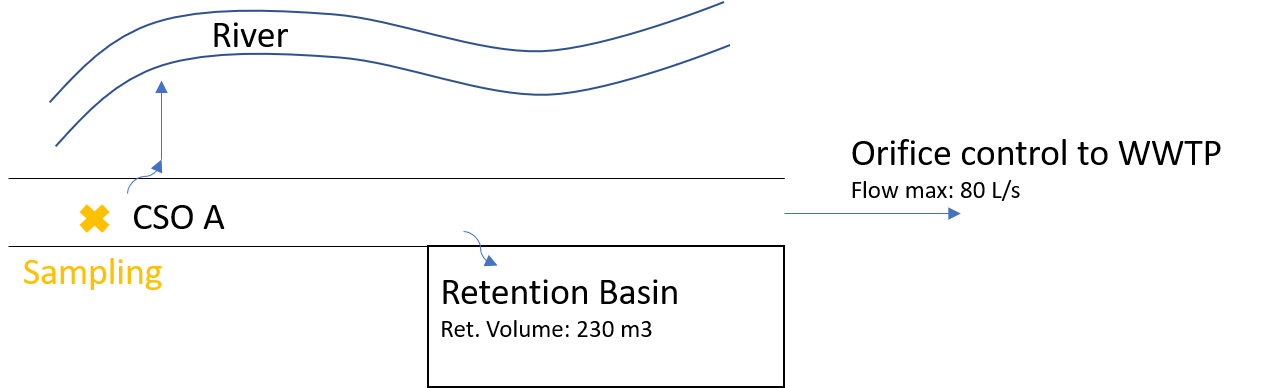


Figure 15: Flow scheme at sampling point in catchment A.

6.2 Catchment B
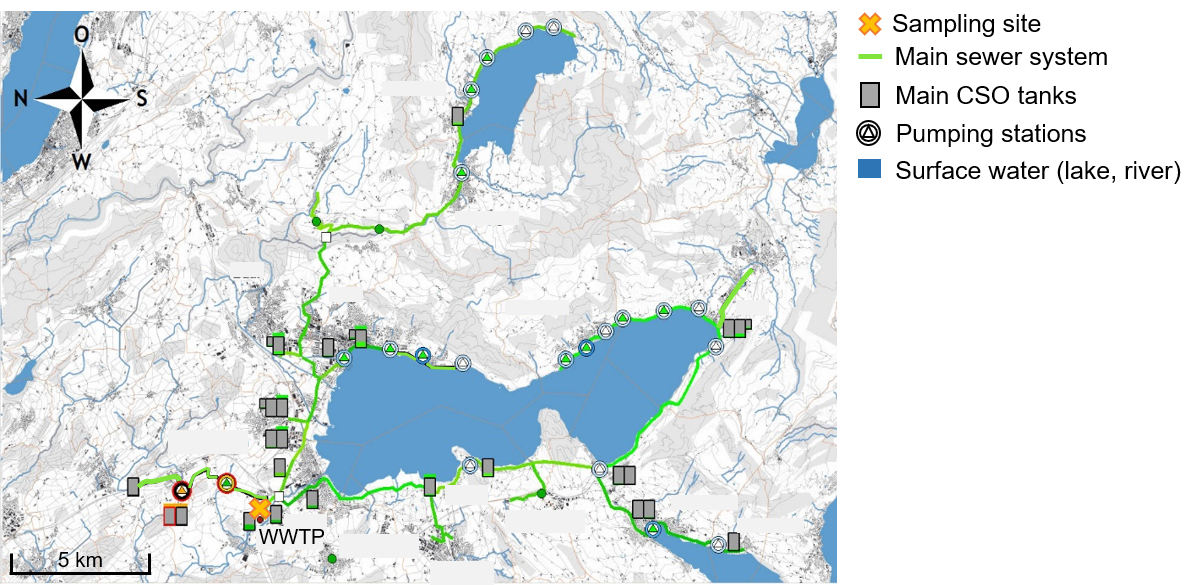


Figure 16: Scheme of catchment B with main sewer system and sampling site (graphic adapted from Michael Arnold).


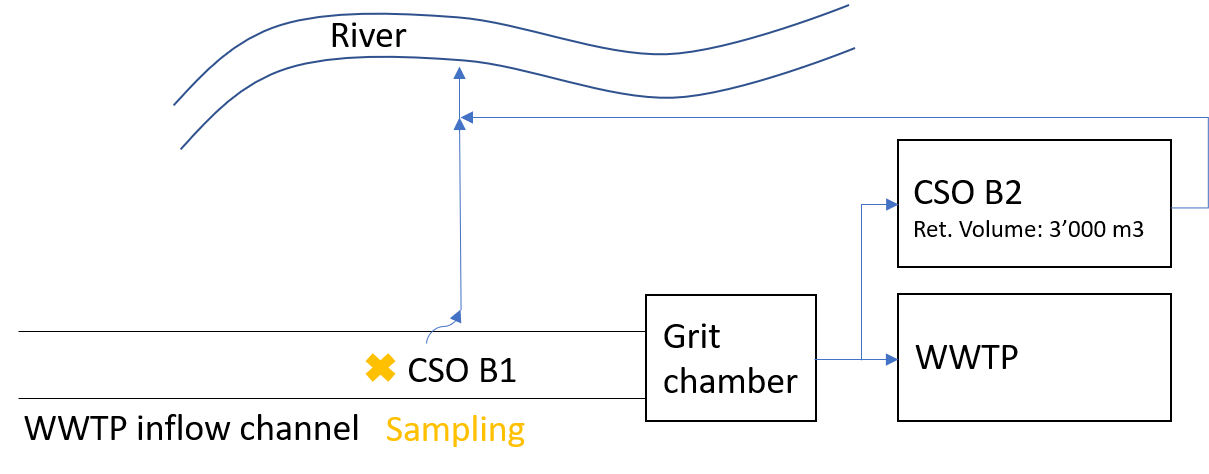


Figure 17: Flow scheme at sampling point in catchment B.

# References

Anliker, S., Loos, M., Comte, R., Ruff, M., Fenner, K. and Singer, H. 2020. Assessing Emissions from Pharmaceutical Manufacturing Based on Temporal High-Resolution Mass Spectrometry Data. Environ Sci Technol 54(7), 4110-4120.
